# Novel pH-sensitive catechol dyes synthesised by a three component one-pot reaction

**DOI:** 10.3389/fchem.2022.1116887

**Published:** 2023-01-10

**Authors:** Juan José Calmels, Leandro Aguilar, Juan Mancebo-Aracil, Gabriel Radivoy, Claudia Domini, Mariano Garrido, Miguel D. Sánchez, Fabiana Nador

**Affiliations:** ^1^ Instituto de Química del Sur (INQUISUR-CONICET)—Grupo de Nanocatálisis y Síntesis Orgánica del Sur Departamento de Química, Universidad Nacional del Sur (UNS), Bahía Blanca, Buenos Aires, Argentina; ^2^ Instituto de Química del Sur (INQUISUR-CONICET), Departamento de Química, Universidad Nacional del Sur (UNS), Bahía Blanca, Argentina; ^3^ Instituto de Física del Sur (IFISUR-CONICET), Departamento de Física, Universidad Nacional del Sur (UNS), Bahía Blanca, Argentina

**Keywords:** catechol, indolizine, dye, pH indicator, nanoparticles

## Abstract

The synthesis and characterisation of new dyes based on indolizines bearing catechol groups in their structure is presented. The preparation was carried out through a simple three component one-pot reaction promoted by CuNPs/C, between pyridine-2-carbaldehyde, an aromatic alkyne and a tetrahydroisoquinoline (THIQ) functionalized with catechol groups. The products were isolated in 30%–34% yield, which was considered more than acceptable considering that the catechol hydroxyl groups were not protected prior to reaction. In view of the colour developed by the products and their response to the acidic and basic conditions of the medium, product **3aa** was studied by UV-Vis and NMR spectroscopies at different pH values. We concluded that product **3aa** suffered two deprotonations at pK_a_ of 4.4 and 9.5, giving three species in a pH range between 2–12, with colours varying from light red to deep orange. The reversibility of the process observed for **3aa** at different pH values, together with its changes in colour, make this new family of products attractive candidates to use them as pH indicators.

## 1 Introduction

Catechol-based materials are a topic of growing interest in recent decades (Yang et al., 2014; Krogsgaard et al., 2016; Forooshani, Lee, 2017; Zhang et al., 2017; Guo et al., 2018; Gallastegui et al., 2019). The application area of these materials is very wide and varied, and it is mainly due to the adhesive and chelating properties of the catechol moiety (Sedó et al., 2013; Ryu et al., 2015; Saiz-Poseu et al., 2019; Zhang et al., 2020). Proof of that is the increasing number of publications on this subject, covering various disciplines such as organic chemistry (Yang et al., 2002; Iacomino et al., 2017; Ji et al., 2019; Mancebo-Aracil et al., 2019; Nador et al., 2021), surface and supramolecular chemistry (Panchireddy et al., 2018; Qiu et al., 2018; Lyu et al., 2019; Souza Campelo et al., 2020), or electrochemistry (Golabi, Nematollahi, 1997; Shahrokhian, Hamzehloei, 2003; Zhao et al., 2012; Toghan et al., 2019). With regard to the use of catechols in optical applications, several examples have been reported where catechol is attached to a fluorescent compound, taking advantage of the synergy between the chelating properties of catechol and the optical properties of the fluorophore to generate new chemosensors (Evangelio et al., 2008; An et al., 2010a; Queirós et al., 2012; Queirós et al., 2014; Yin et al., 2014; Queirós et al., 2015; Monteiro-Silva et al., 2021), and materials (Kaushik et al., 2015; Nador et al., 2018a; Nador et al., 2018b; Mancebo-Aracil et al., 2019). Catechols have also been used as pendant groups in fluorescent polymers to improve their adhesion (Zhang et al., 2016; Gapin et al., 2020; Zhan et al., 2021). Moreover, a large number of articles have reported the use of the catechol moiety in dye-sensitized solar cells, taking advantage of the binding properties of catechol as anchoring group, through bidentate mononuclear chelating and/or bidentate binuclear bridging linkages on the TiO_2_ surface (An et al., 2010b; Ooyama et al., 2014).

More specifically, catechols are present in water-soluble plant pigments, such as anthocyanins, which have been exploited in natural hybrid pigments with pH-sensing activity, (Ogawa et al., 2017; Li et al., 2019), colourimetric determination of various metal cations and for colour development in food applications (Tang, Giusti, 2020; Torrini et al., 2022). In terms of commercial catechol dyes, either as single molecules or embedded in different materials, the most widely used in colourimetric applications are pyrocatechol violet, pyrogallol red, bromopyrogallol red and alizarin red. Some more prominent uses of these catechol dyes include their use as colourimetric sensors (Gaidamauskas et al., 2011; Sasaki et al., 2019; Wang et al., 2020; Estévez et al., 2021; Lyu et al., 2021) and as pH-indicator dyes (Ivanov, Kochelayeva, 2006; Lee, Lee, 2014; Szadkowski et al., 2022).

From the above, the importance of catechols in optical applications is more than evident. However, many of the examples published so far reporting the incorporation of the catechol moiety in more complex structures, or even for the synthesis of catechol-based dyes, are not straightforward and require several reaction steps, including catechol protection/deprotection reactions. To the best of our knowledge, there are no examples in the literature where catechol-based dyes with varied substitution pattern are generated through a multicomponent one-pot reaction. Even less if hydroxyl groups of the catechol functionality are not protected during the synthetic process.

**SCHEME 1:**
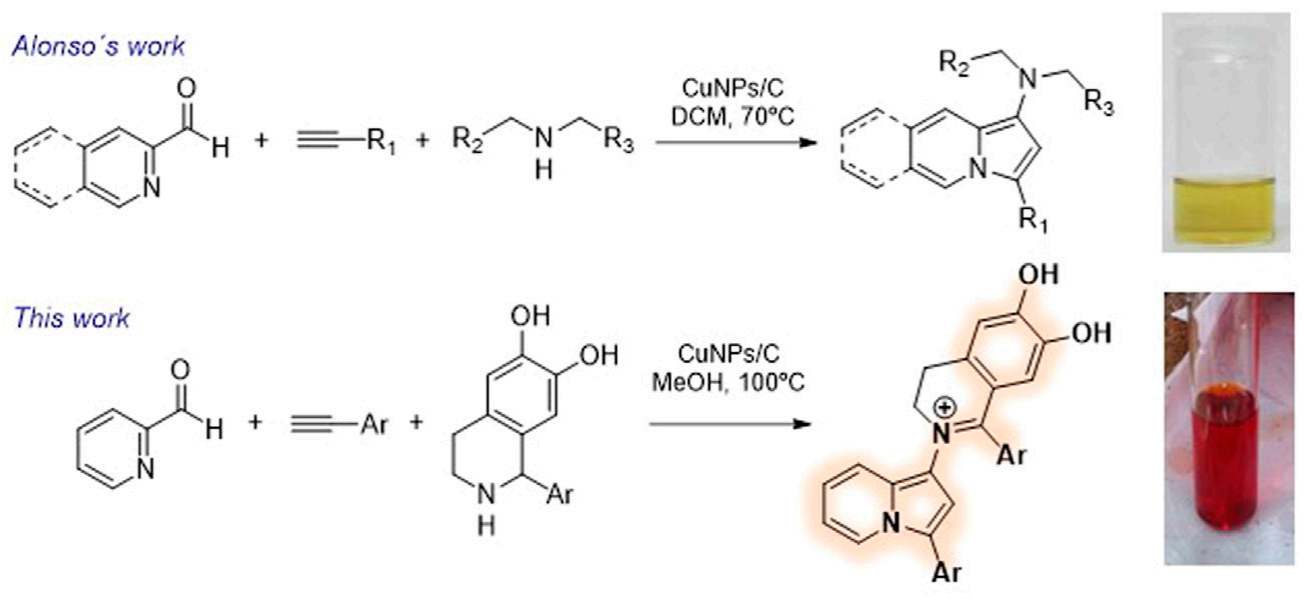
Synthesis of indolizines by three-component reactions.

On the other hand, Alonso and co-workers reported a multicomponent reaction between pyridine-2-carbaldehyde derivatives, secondary amines and terminal alkynes to produce indolizines (Scheme 1) (Albaladejo et al., 2013; Albaladejo et al., 2015). It is important to note that most of these indolizines presented a yellow colouring and that after treatment with AcOH turned to red/violet, due to the formation of a dye with D-A-π-A configuration, through a transmutation process from the starting material (Albaladejo et al., 2018). Inspired by these works, we decided to perform a similar approach but using 1, 2, 3, 4-tetrahydroisoquinolines (THIQs) as secondary amines bearing a catechol moiety in their structure. To our delight, the corresponding indolizines containing the catechol skeleton, and with an intense red colour, were obtained through a simple multicomponent process catalysed by copper nanoparticles supported on activated charcoal (CuNPs/C) (Scheme 1).

Here we describe a three component one-pot reaction, for the synthesis of new indolizine dyes bearing a catechol moiety with varied substitution pattern, which were obtained in moderate to good yields. Because of the intense red colour of these structures, the UV-Vis absorption properties were studied, as well as their use as pH indicators.

## 2 Materials and methods

### 2.1 General

Anhydrous tetrahydrofuran was freshly distilled from sodium/benzophenone ketyl. DCM and MeOH solvents were previously distilled. All starting materials were of the best available grade (Merck, Fluka, Anedra, AK Scientific, Alpha Aesar) and were used without further purification. Commercially available copper (II) chloride dihydrate was dehydrated upon heating in oven (150°C, 45 min) prior to use for the preparation of CuNPs. Column chromatography was performed with Merck silica gel 60 (.040–.063 μm, 240–400 mesh) and DCM/MeOH as eluent. Reactions were monitored by thin-layer chromatography on silica gel plates (60F-254) visualized under UV light and/or using ninhydrine or FeCl_3_ as revelators.

All crudes and products containing THIQ were properly protected from light. ^1^H NMR and ^13^C NMR were measured on a Bruker AV-300 spectrometer with chemical shifts reported in ppm (in DMSO-d_6_, MeOD-d_4_ or CDCl_3_). Infrared spectra were collected on a Nicolet iS50 FTIR spectrophotometer in attenuated total reflectance mode (ATR-FTIR) with a Smart iTR (Thermo Fisher Scientific) single reflection diamond (42° angle, sampling área, 1.5 mm). Spectra were collected on a range wavelength from 4,000 to 650 cm-1, 256 scans and 8 cm-1 resolution. ESI-MS were analyzed on an Agilent Model 1,100 Series High Performance Liquid Chromatograph simultaneously coupled to a UV-visible variable wavelength detector and an ion trap analyzer mass spectrometer (Agilent Model 1,100 Series LC/MSD Trap SL). Microwave reactions were performed with a microwave oven (CEM Discover®) with a continuous focused microwave power delivery system in a pressure glass vessel (10 mL) sealed with a septum under magnetic stirring.

To achieve pH analysis, a Britton-Robinson buffer solution was employed. Fluorescence measurements were performed at room temperature on a Shimadzu and RX was used as a standard for the determination of fluorescence quantum yields.

The surface composition and oxidation state of the nanocatalysts were determined by X-ray photoelectron spectroscopy (XPS) in a PHI 548 spectrometer, using the non-monochromatic Mg Kα radiation at 250 W and 20 mA. The resolution spectra were taken at 50 eV of pass energy, giving an energy resolution of ± .5 eV. The operation base pressure was better than 5 × 10–9 Torr. The C-C bond binding energy was taken as an internal charge reference fixed at 284.8 eV. The signal deconvolution was made using Shirley-type background subtraction and the sum of Gaussian-Lorentzian functions as a peaks model. The atomic ratio estimations were done relating the peak areas after the background subtraction and corrected relative to the corresponding atomic sensitivity factors to an approximated absolute error of 20%. The x-ray peak satellites were numerically subtracted.

### 2.2 Synthesis and characterization of THIQs 2a, 2b and 2c

For the synthesis of THIQ **2a**, **2b** and **2c** an adaptation of the procedure published by Xiang was performed (Yang et al., 2019). Dopamine hydrochloride (3 mmol, 569 mg) was placed in a dry flask under N_2_ atmosphere and phosphate buffer at pH 6 (35 mL) was added. Subsequently aldehyde (5 mmol, 690 mg) was added. The suspension was heated at 40°C for 6 h. After this time, the suspension was filtered and placed in a separation funnel. Washes were made with AcOEt (4 × 5 mL) and the aqueous phase was evaporated. The crude was purified, previous pelleting, by column chromatography on flash silica gel with MeOH/DCM as mobile phase to afford the corresponding THIQ.

**1-(3,4-dihydroxyphenyl)-1,2,3,4-tetrahydroisoquinoline-6,7-diol** (**2a**). The reaction mixture was purified by column chromatography with 9/1 to 7/3 gradient of DCM/MeOH, yielding THIQ **2a** in 64%. Yellow-brownish solid, R_f_: .3 (DCM:MeOH, 8:2). ^1^H NMR (300 MHz, DMSO-d_6_): 6.78 (d, *J* = 8.0 Hz, 1H); 6.72 (s, 1H); 6.64 (d, *J* = 8.1 Hz, 1H); 6.58 (s, 1H); 6.12 (s, 1H); 5.27 (s, 1H); 3.27–3.22 (m, 2H); 3.12–2.97 (m, 1H); 2.81–2.74 (m, 1H), OH and NH n.d. ^13^C NMR (75 MHz, DMSO-d_6_): 146.0 (C); 145.2 (C); 145.1 (C); 143.9 (C); 128.7 (C); 123.5 (C); 123.0 (C); 120.9 (CH); 116.9 (CH); 115.4 (CH); 114.9 (CH); 114.3 (CH); 58.3 (CH); 40.3 (CH_2_); 24.6 (CH_2_). IR-ATR (cm^−1^): 3,280-3,050, 1,613, 1,525, 1,233, 1,186.

**1-phenyl-1,2,3,4-tetrahydroisoquinoline-6,7-diol** (**2b**). The reaction mixture was purified by column chromatography with 9/1 to 7/3 gradient of DCM/MeOH, yielding THIQ **2b** in 74%. Light yellow-brownish solid, R_f_: .32 (DCM:MeOH, 8.5:1.5). ^1^H NMR (300 MHz, MeOD-d_4_): 7.46–7.45 (m, 3H); 7.40–7.38 (m, 2H); 6.69 (s, 1H); 6.19 (s, 1H); 5.55 (s, 1H); 3.51–3.39 (m, 2H); 3.19–2.96 (m, 2H), OH and NH n.d. ^13^C NMR (75 MHz, MeOD-d_4_): 147.0 (C); 145.7 (C); 138.4 (C); 130.9 (2xCH); 130.6 (CH); 130.2 (2xCH); 124.6 (C); 123.5 (C); 115.9 (CH); 115.4 (CH); 60.7 (CH); 41.4 (CH_2_); 25.7 (CH_2_). IR-ATR (cm^−1^): 3,300-3,050, 2,939, 1,614, 1,509, 1,284, 1,183, 1,109.

**1-methyl-1,2,3,4-tetrahydroisoquinoline-6,7-diol** (**2c**). The reaction mixture was purified by column chromatography with 9.5/0.5 to 8/2 gradient of DCM/MeOH, yielding THIQ **2c** in 59%. Brown solid, R_f_: .24 (DCM:MeOH, 8.5:1.5). ^1^H NMR (300 MHz, MeOD-d_4_): 6.61 (s, 1H); 6.55 (s, 1H); 4.41 (q, *J* = 6.8 Hz, 1H); 3.50–3.42 (m, 1H); 3.33–3.24 (m, 1H); 3.01–2.80 (m, 2H); 1.58 (s, *J* = 6.8 Hz, 3H), OH and NH n.d. ^13^C NMR (75 MHz, MeOD-d_4_): 146.5 C; 145.9 (C); 125.3 (C); 123.3 (C); 116.1 (CH); 113.4 (CH); 52.3 (CH); 40.9 (CH_2_); 25.7 (CH_2_), 19.7 (CH_3_). IR-ATR (cm^−1^): 3,300-3,050, 2,939, 1,614, 1,509, 1,284, 1,183, 1,109.

### 2.3 Synthesis and characterization of THIQs 2d

For the synthesis of THIQ **2d**ays, the procedure published by Capretta was performed (Awuah, Capretta, 2010). 2-(3-methoxyphenyl)ethan-1-amine (1 mmol), benzaldehyde (1.2 mmol), TFA (8 mmol) and toluene (1 mL) were placed in a microwave vial, which was then capped and irradiated in a microwave for 30 min at 140°C. The solvent was evaporated under reduced pressure and the crude was suspended in cold water (3 mL). Then, a solution of NaOH 2 M was added dropwise to reach pH 8. In this point a yellowish white suspension was observed. It was extracted with DCM (3 × 6 mL) and dried over MgSO_4_. Finally, it was purified by flash column chromatography on silica gel.

**6-methoxy-1-phenyl-1,2,3,4-tetrahydroisoquinoline** (**2** **d**ays). The reaction mixture was purified by column chromatography with DCM to 9.8/0.2 gradient of DCM/MeOH, yielding THIQ **1d**ay in 38%. Yellowish pink solid, R_f_: .42 (DCM:MeOH, 9.5:0.5). ^1^H NMR (300 MHz, MeOD-d_4_): 7.34–7.26 (m, 3H); 7.24–7.21 (m, 2H); 6.71 (s, 1H); 6.63–6.57 (m, 2H); 5.00 (s, 1H); 3.75 (s, 3H, OCH_3_); 3.22–3.13 (m, 1H); 3.05–2.93 (m, 2H); 2.85–2.76 (m, 1H), NH n.d. ^13^C NMR (75 MHz, MeOD-d_4_): 159.6 C; 145.5 C; 137.6 C; 131.0 C; 130.1 (3xCH); 129.4 (2xCH); 128.5 (CH); 114.2 (CH); 113.2 (CH); 62.4 (CH); 55.6 (CH_3_O); 42.5 (CH_2_); 30.2 (CH_2_). IR-ATR (cm^−1^): 3,246, 3,062, 2,906, 2,832, 1,602, 1,499, 1,275, 1,043.

### 2.4 Preparation and characterization of CuNPs/C catalyst

Anhydrous copper (II) chloride (134 mg, 1 mmol) was added to a suspension of lithium (21 mg, 3 mmol) and 4.4′-di-tert-butylbiphenyl (DTBB, 27 mg, .1 mmol) in THF (2 mL) at room temperature under a nitrogen atmosphere (Alonso et al., 2010; Alonso et al., 2011). The reaction mixture, which was initially dark blue, rapidly changed to black, indicating that the suspension of copper nanoparticles was formed. This suspension was diluted with THF (6 mL) followed by the addition of the activated charcoal (800 mg). The resulting mixture was stirred for 1 h at room temperature, quenched with water and then filtered. The solid was successively washed with EtOH (7 mL) and dried under vacuum.

### 2.5 Procedure for the one-pot three component synthesis of products 3 and their characterization

Pyridine-2-carbaldehyde (.44 mmol), THIQ (.2 mmol) and alkyne (.44 mmol) were added to a reactor sealed tube containing CuNPs/C (45 mg, ca. 10 mol%) and MeOH (1.5 mL). The reaction mixture was warmed to 100°C for 24 h and was monitored by TLC. After completion, the reaction was filtered on celite and washed with MeOH. Finally, the product was purified by chromatographic column using flash silica as stationary phase. It was compacted with a 3% Et_3_N/DCM solution, washed with DCM and the sample was directly seeded. The elution gradient employed was DCM to 20% MeOH/DCM to afford the corresponding product **3**.

**1-(3,4-dihydroxyphenyl)-6,7-dihydroxy-2-(3-phenylindolizin-1-yl)-3,4-dihydroisoquinolin-2-ium** (**3aa**). The yield of the isolated product was 31%. Dark red solid, R_f_: 0.54 (DCM:MeOH, 8.5:1.5). ^1^H NMR (300 MHz, DMSO-d_6_): 9.90 (br s, 1H, OH); 9.45 (br s, 1H, OH); 8.32 (d, *J* = 7.2 Hz, 1H); 7.60–7.34 (m, 8H); 7.04–6.81 (m, 4H); 6.78–6.65 (m, 4H); 4.49–4.44 (m, 2H); 3.29–3.25 (m, 2H). ^13^C NMR (75 MHz, DMSO-d_6_): 171.9 (C); 155.9 (C); 148.7 (C); 144.7 (C); 144.6 (C); 133.7 (C); 130.2 (C); 129.2 (2xCH); 127.9 (CH); 127.8 (2xCH); 125.9 (C); 124.1 (C); 122.8 (CH); 122.7 (CH); 121.9 (C); 120.5 (CH); 120.1 (CH); 119.0 (C); 118.3 (C); 117.5 (CH); 116.6 (CH); 115.3 (CH); 115.1 (CH); 112.4 (CH); 110.8 (CH); 55.3 (CH_2_); 25.4 (CH_2_). IR-ATR (cm^−1^): 3,365-3,122, 3,048, 1,595, 1,513, 1,294, 1,193. LRESI-MS m/z 463.1 [M]+ (calcd for C_29_H_23_N_2_O_4_, 463.2).

**1-(3,4-dihydroxyphenyl)-6,7-dihydroxy-2-(3-(p-tolyl)indolizine-1-yl)-3,4-dihydroisoquinolin-2-ium** (**3ab**). The yield of the isolated product was 31%. Dark red solid, R_f_: 0.54 (DCM:MeOH, 8.5:1.5). ^1^H NMR (300 MHz, DMSO-d_6_): 8.26–8.13 (m, 1H); 7.61–7.07 (m, 7H); 7.04–6.20 (m, 11H); 4.54–4.25 (m, 2H); 3.30–3.01 (m, 2H); 2.34 (s, 3H). ^13^C NMR (75 MHz, DMSO-d_6_): 169.1 (C); 161.7 (C); 148.2 (C); 145.6 (C); 144.6 (C); 137.2 (C); 135.2 (C); 129.7 (2xCH); 127.7 (C); 127.7 (2xCH); 127.5 (C); 126.0 (C); 123.8 (C); 122.6 (C); 122.6 (CH); 122.5 (CH); 119.4 (CH); 118.7 (C); 117.8 (CH); 117.5 (CH); 116.6 (CH); 115.8 (CH); 115.0 (CH); 112.1 (CH); 110.7 (CH); 54.7 (CH_2_); 26.2 (CH_2_); 20.8 (CH_3_). IR-ATR (cm^−1^): 3,350-3,120, 3,077, 2,958, 1,593, 1,510, 1,284, 1,157. LRESI-MS m/z 477.2 [M]+ (calcd for C_30_H_25_N_2_O_4_, 477.2).

**1-(3,4-dihydroxyphenyl)-6,7-dihydroxy-2-(3-(4-methoxyphenyl)indolizin-1-yl)-3,4-dihydroisoquinolin-2-ium** (**3ac**). The yield of the isolated product was 34%. Dark red solid, R_f_: 0.6 (DCM:MeOH, 8:2). ^1^H NMR (300 MHz, DMSO-d_6_): 8.20 (d, *J* = 7.1 Hz, 1H); 7.53 (d, *J* = 9.1 Hz, 1H); 7.47–7.36 (m, 2H); 7.13–7.02 (m, 3H); 6.77–6.72 (m, 6H); 6.72–6.65 (m, 5H); 4.43–4.41 (m, 2H); 3.80 (s, 3H); 3.22–3.20 (m, 2H). ^13^C NMR (75 MHz, DMSO-d_6_): 170.2 (C); 159.0 (C); 158.9 (C); 148.4 (C); 145.1 (C); 144.6 (C); 134.5 (C); 129.4 (2xCH); 125.5 (C); 123.9 (C); 122.6 (CH); 122.6 (CH); 122.6 (C); 122.4 (C); 119.4 (CH); 119.0 (CH); 118.3 (C); 117.5 (CH); 116.5 (CH); 115.6 (CH); 115.1 (CH); 114.7 (2xCH); 112.1 (CH); 110.3 (CH); 55.3 (CH_3_O); 55.0 (CH_2_); 25.8 (CH_2_). IR-ATR (cm^−1^): 3,370-3,120, 3,075, 1,601, 1,509, 1,283, 1,172. LRESI-MS m/z 493.2 [M]+ (calcd for C_30_H_25_N_2_O_5_, 493.2).

**6,7-dihydroxy-1-phenyl-2-(3-phenylindolizin-1-yl)-3,4-dihydroisoquinolin-2-ium** (**3ba**). The yield of the isolated product was 30%. Dark red solid, R_f_: 0.79 (DCM:MeOH, 8.5:1.5). ^1^H NMR (300 MHz, DMSO-d_6_): 8.27 (d, *J* = 7.2 Hz, 1H); 7.71 (d, *J* = 8.8 Hz, 1H); 7.50–7.48 (m, 5H); 7.45–7.37 (m, 7H); 7.12–7.05 (m, 1H); 6.96–6.89 (m, 2H); 6.69 (dd, *J* = 6.8 and 6.9 Hz, 1H); 6.60 (s, 1H); 4.58–4.53 (m, 2H); 3.08–3.01 (m, 2H). ^13^C NMR (75 MHz, DMSO-d_6_): 173.0 (C); 156.1 (C); 144.8 (C); 133.6 (C); 131.6 (C); 131.0 (CH); 130.0 (C); 129.3 (2xCH); 129.2 (2xCH); 128.1 (2xCH); 128.0 (CH); 127.8 (2xCH); 126.2 (C); 124.1 (C); 122.8 (CH); 120.4 (CH); 120.2 (CH); 119.1 (C); 117.6 (C); 116.6 (CH); 115.4 (CH); 112.5 (CH); 111.0 (CH); 55.4 (CH_2_); 25.3 (CH_2_). IR-ATR (cm^−1^): 3,300-3,120, 3,045, 1,597, 1,536, 1,299.

## 3 Results and discussion

### 3.1 Optimization of reaction conditions

We decided to begin our studies by using pyridine-2-carbaldehyde, phenylacetylene and a tetrahydroisoquinoline **2a** (THIQ **2a**) as model compounds (Table 1). The THIQ 2a was synthesized *via* a Pictet-Spengler cyclization between dopamine hydrochloride and 3,4-dihydroxybenzaldehyde following a previously reported procedure (see experimental and Section 1 in Supplementary Material S1) (Yang et al., 2019). Table 1 shows the different reaction conditions tested during the optimization process. All reactions were performed in the presence of the CuNPs/C catalyst prepared by the addition of the activated charcoal to a suspension of freshly prepared copper nanoparticles (CuNPs), following the methodology already reported by our group (see experimental section) (Nador et al., 2013; Nador et al., 2021). As shown in entries 1 and 2, as starting conditions an equimolar amount of pyridine-2-carbaldehyde, phenylacetylene and THIQ **2a** were dissolved in DCM or MeOH, and heated at 70°C under air atmosphere. No reaction progress was observed after 24 h in any of both cases. A 5-fold increase in the amount of the CuNPs catalyst did not produce any improvement when DCM was used as the reaction solvent (entry 3). However, by changing the solvent to MeOH, an intense dark red colouring was observed in the reaction mixture after 4 h. This was confirmed by TLC as the formation of a dark red spot with higher R_f_ value than **2a**. Analysis by ^1^H NMR revealed the formation of product **3aa** with 6% conversion (entry 4). Under the same conditions but using water, EtOH or MeCN as the solvent, no product formation was noted (entries 5-7). The same reaction under solventless conditions generated a faint red spot in TLC, however mainly the starting THIQ **2a** was observed by ^1^H NMR analysis (entry 8). When the reaction conditions detailed in entry 4 were reproduced but under heating at 100°C in a closed Schlenk tube, the reaction progress increased to 21% conversion into **3aa** (entry 9). Nevertheless, although virtually no starting aldehyde was observed, some of the starting THIQ **2a** remained unreacted. Other nanocatalysts such as CuNPs/ZnO, CuNPs/TiO_2_ and CuNPs/ZY were tested under the reaction conditions shown in entry 9 giving very low conversions (0%–5%) to the desired product (data not shown). Based on this result, we decided to try the reaction by using an excess of aldehyde and alkyne (entry 10). In this case, no unreacted THIQ **2a** was observed, improving the conversion to **3aa** (38%). Interestingly, an even larger excess of alkyne and aldehyde increased the conversion to **3aa** up to 58% (entry 11). A further increase in alkyne and aldehyde excess, as well as longer reaction times, did not improve the conversion, leading to the formation of undesired side products. Also, an excess of the catalyst loading (20 mol%), under the same conditions, did not afford an increment on the conversion to the desired adduct (entry 12). Furthermore, the use of an O_2_ atmosphere under entry 11 conditions, proved counterproductive, leading to almost no-conversion to **3aa** together with the formation of a complex mixture of by-products (entry 13). Then, in order to evaluate the influence of the nature of the solvent in the reaction course, we tested the methodology under the same conditions as those reported in entry 11 but using MeCN, DMSO, DMF, toluene and EtOH as the reaction solvent. As shown in entries 14-18, under these conditions no conversion to the desired product **3aa** was observed. An additional experiment using CuCl_2_ as copper source was performed, giving no reaction product (entry 19). Finally, the reaction was carried out under the optimised conditions of entry 11 but in the absence of copper source, showing no product formation.

**TABLE 1:** Optimisation of reaction conditions.

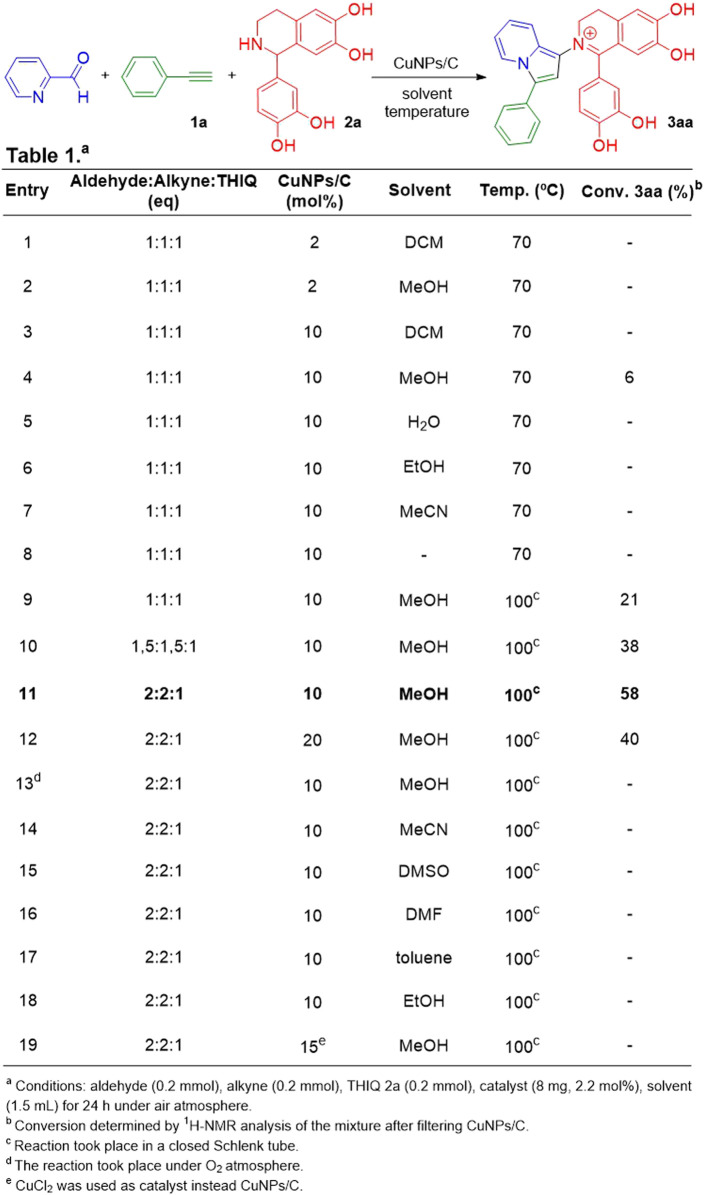

Regarding the isolation of **3aa**, even when we considered it important to avoid the purification by column chromatography, due to the presence of catechol groups that strongly adsorb to the stationary phase (Al_2_O_3_ or SiO_2_), all attempts to wash the sample with low polarity solvents or recrystallisations to obtain the pure product were unsuccessful. On the other hand, when we tried to carry out the purification through flash column chromatography with silica gel as the stationary phase, the product **3aa** underwent a ring opening process giving a propargylamine derivative as main product (Nador et al., 2013). In view of this, we decided to deactivate the silica gel by using DCM dopped with Et_3_N (3%) as the eluent, that allowed us to isolate the product **3aa** without decomposition after washing it with a .1 M HCl solution. The product was thoroughly characterized by NMR, ESI-MS and IR (see experimental and S2 sections). After sample work-up, we confirmed the presence of a halide counterion in the product by Beilstein’s and AgNO_3_ tests, which was assigned to chloride ion (see S2.2 section).

### 3.2 Scope of the reaction

**FIGURE 1:**
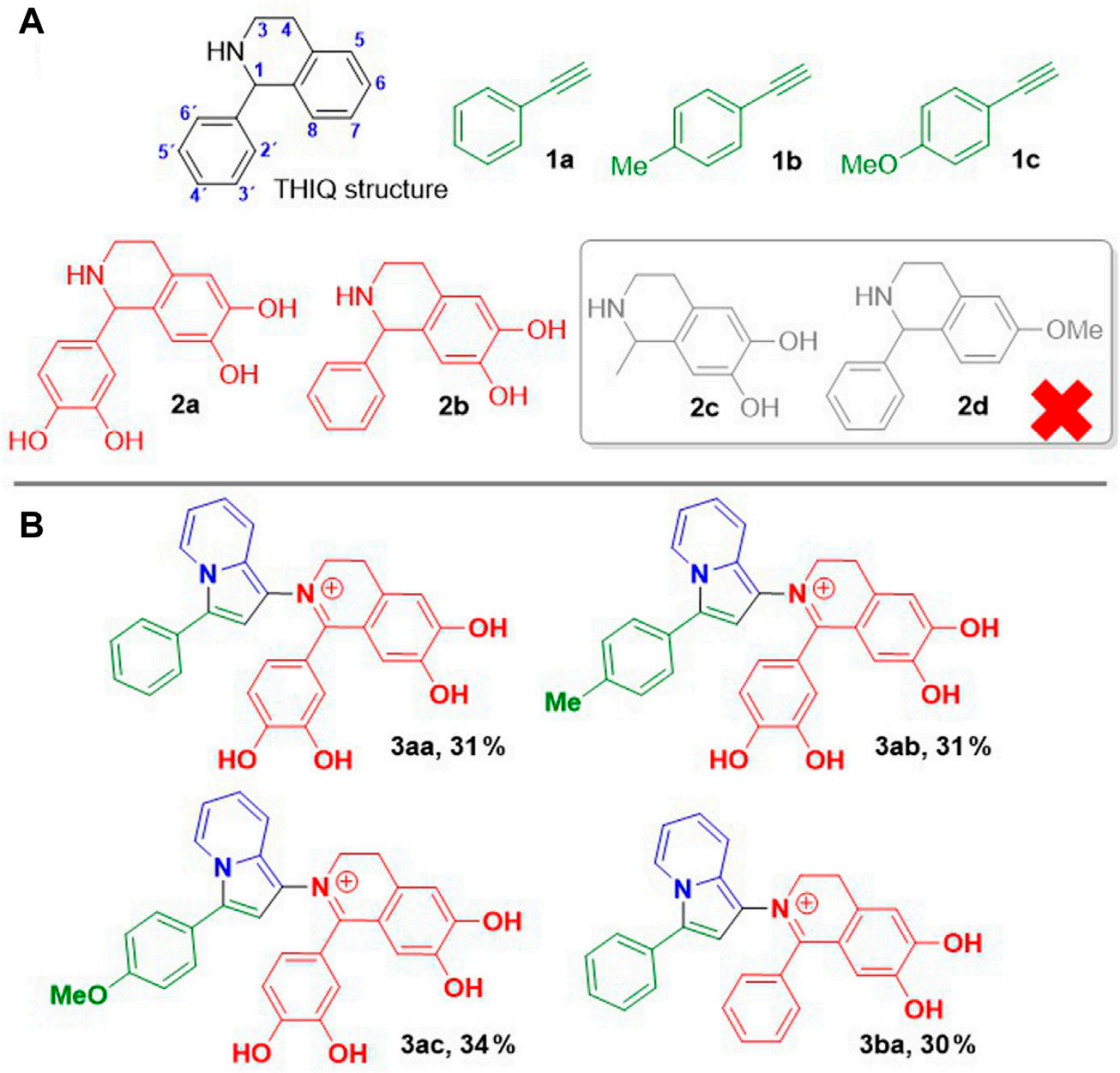
**(A)** Structures of synthesized THIQs and alkynes used as starting materials. **(B)** Structure and yields for products **3** obtained through three component coupling.

Then, under the optimized conditions, the scope of the three component reaction was studied. We decided to start working with pyridine-2-carbaldehyde and THIQ 2a, varying only the starting alkyne. Aromatic alkynes proved to be the most convenient partners; both phenylacetylene, 4-methylphenylacetylene and 4-methoxyphenylacetylene gave the products 3aa, 3ab and 3ac, respectively, in 31%–34% yield after purification following the same work-up described above for 3aa (Figure 1B).

Since the process is a three component one-pot reaction, and works with unmasked catechols that could strongly adsorb on the silica gel stationary phase, we considered that the isolated yields obtained are good enough. When we tried the reaction with 4-ethynyl-*N*,*N*-dimethylaniline, the intense red colouring was not observed, instead a brown solid was obtained which was assigned to an open chain propargyl-type structure. On the other hand, the three component reaction failed when carried out with an electron-deficient aromatic alkyne such as 2-ethynylpyridine, or with functionalized alkynes such as propargyl alcohol, propargylamine or 2-ethynylcyclohexene.

Next, the scope of the reaction was studied by using different THIQs as starting amines. We focused on the formation of the iminium bond (C=N^+^) which leads to an extended conjugation throughout the whole product structure and consequently developing the observed red colouring. We started our study by using THIQ **2b**, which has no OH groups in the aryl ring located at C1 of the THIQ skeleton (see Figure 1A). When the three component reaction was carried out with phenylacetylene, pyridine-2-carbaldehyde and THIQ **2b**, product **3ba** as a reddish solid was formed and isolated in 30% yield. In this case two red spots were observed in the TLC, which after work-up contributed to the same product (see discussion below). Subsequently, we decided to test the same reaction by using a starting THIQ substituted with an alkyl group at C1. For this, the reaction was carried out with phenylacetylene, pyridine-2-carbaldehyde and THIQ **2c**. Although the reaction turned bright red within a few hours, we discarded to carry out the product isolation and purification because numerous coloured side products were observed by TLC. We wondered whether the OH groups at positions 6 or 7 of the starting THIQ were relevant to the formation of product **3**. For this purpose, the three component reaction was carried out with THIQ **2d**ays. Here again, as observed for THIQ **2c**, although the reaction acquired a reddish colouration after a few hours, analysis by TLC of the crude reaction revealed the formation of a complex mixture of by-products.

### 3.3 Proposed mechanism

**SCHEME 2:**
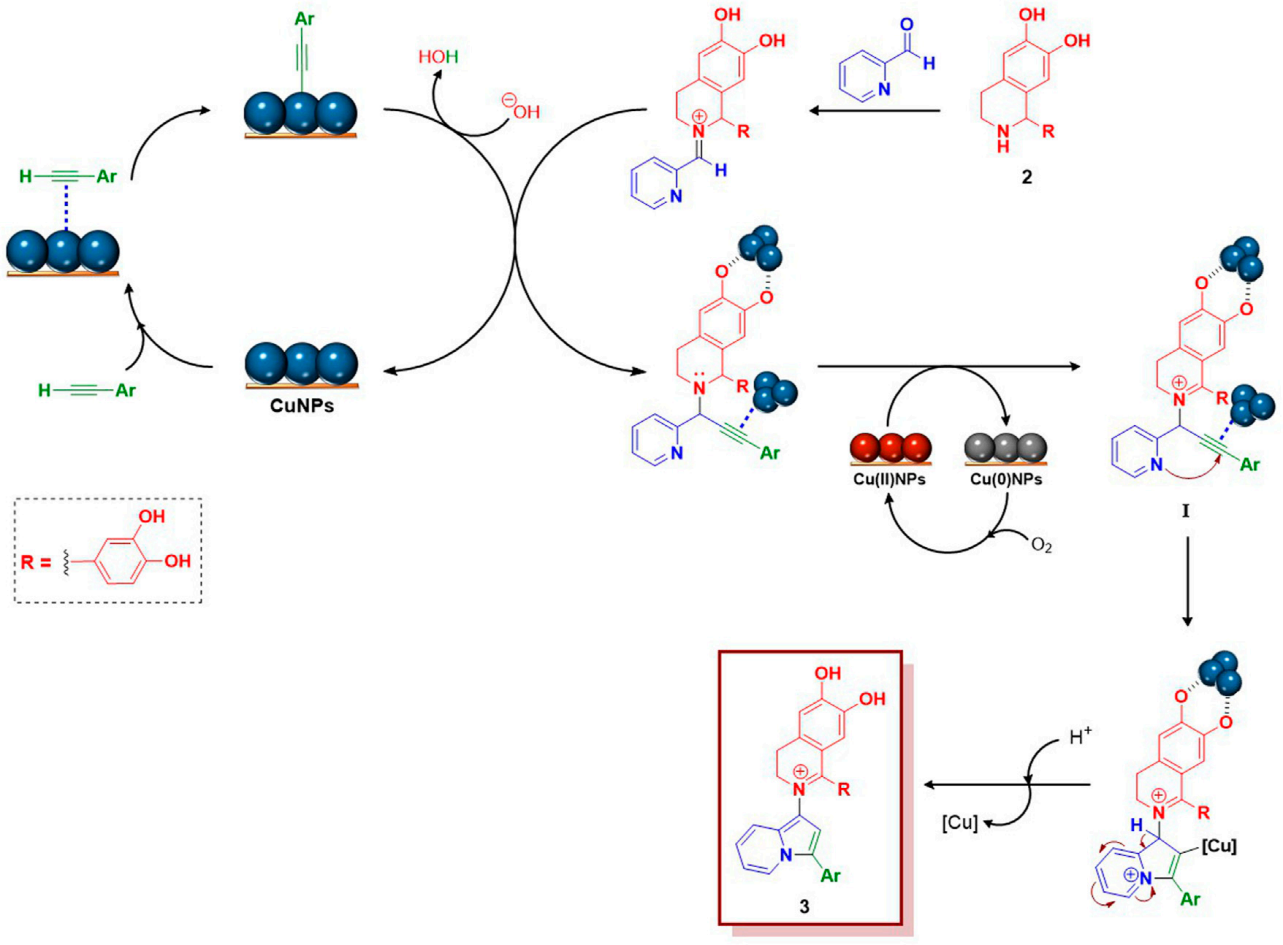
Reaction mechanism proposed for the synthesis of product 3.

Regarding the possible mechanism for this reaction, based on related work previously published by Alonso’s group (Albaladejo et al., 2013; Albaladejo et al., 2015), the formation of an iminium ion by condensation between the aldehyde and the THIQ, followed by addition of the CuNPs-activated alkyne to form a propargylamine is proposed (Scheme 2). Subsequently, a copper-promoted oxidation of the THIQ ring at the benzylic position, leading to an iminium ion, is suggested (intermediate **I**). A similar oxidation of the THIQ skeleton has been previously reported in the oxidative coupling of *N*-aryl-1.2,3,4-tetrahydroisoquinolines with different nucleophiles, using copper-based catalysts and several oxidants (Boess et al., 2012; Willms et al., 2018; Boess et al., 2020; Bjerg et al., 2022). Moreover, commonly, metal-catalysed oxidative coupling reactions with tertiary amines have been also proposed to proceed *via* iminium ion species (Murahashi et al., 2003; Yoo et al., 2010). Finally, as shown in Scheme 2, a copper-promoted cycloisomerisation of the resulting intermediate (**I**), bearing an acetylenic group with enhanced electrophilicity due to both coordination with the copper catalyst and the proximity of the iminium ion, could be proposed as a very likely reaction pathway, to give product **3**.

With respect to the oxidation-promoting species, from the results obtained by X-ray photoelectron spectroscopy (XPS) analysis of the CuNPs/C catalyst, it was concluded that the surface of the fresh catalyst consisted mainly of Cu(0) and Cu(II) assigned to metallic copper and CuO respectively (for a more detailed discussion about XPS analysis, see Section 3 in the Supplementary Material S1).

Thus, the oxidation of the propargylamine intermediate to give a conjugated iminium ion **I** (Scheme 2), through a copper-promoted redox catalytic process, is very plausible. On the other hand, the formation of hemiaminal ether species in the oxidative coupling of THIQs, acting as a reservoir for the formation of the corresponding iminium ion, has been well documented (Catino et al., 2006; Boess et al., 2011; Boess et al., 2012). In this sense, even when we were not able to detect any hemiaminal ether species during the course of the reaction, the formation of such intermediate can not be disregarded. In this respect, the use of MeOH as the solvent in our reaction was demonstrated to be mandatory to assist the formation of the indolizine products **3**. As commented above, the reaction failed when MeCN, DMSO, DMF, Toluene and even EtOH were used as solvents, which would be in line with this assumption.

In addition, post-reaction XPS analysis of the catalyst was also performed. It was concluded that the reacted catalyst would be covered by the reaction product **3** and the starting THIQ **2**, since a marked decrease in the intensity of the peaks corresponding to copper, together with the appearance of nitrogen species, was observed in the XPS spectra (see Section 3 in Supplementary Material S1). These results could be associated with the chelating ability of the catechols towards metals such as copper.

### 3.4 Study of product 3aa by UV-Vis spectroscopy

The product **3aa** was then studied by UV-Vis spectroscopy in different solvents. Supplementary Figure S39 shows the UV-Vis spectra for the product in MeOH, AcOEt, DMSO, and MeCN, revealing three main bands. One band centered about 327 nm was detected for **3aa** in all the solvents.

The second absorption band appeared around 384–406 nm, with maxima at lower wavelengths for the solvents with higher polarity (DMSO and MeCN). The third band was located about 465–485 nm, and showed a redshift for MeOH and AcOEt, with respect to the solvents with higher polarity. In general, the product dissolved in MeOH and AcOEt showed higher molar absorptivity than in the other solvents. Likewise, the fluorescence of **3aa** in different solvents was studied. It was found that in DMSO and MeCN the fluorescence was low, whereas no fluorescence was observed in the other solvents (Supplementary Figure S40).

**FIGURE 2:**
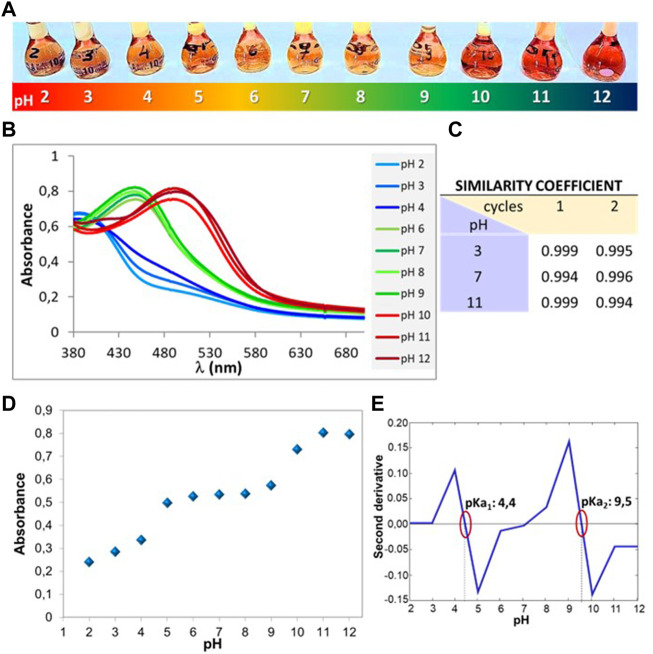
**(A)** Aqueous solutions of **3aa** at different pH. **(B)** UV-Vis spectra of **3aa** at different pH. **(C)** Table with similarity coefficient. **(D)** Absorbance measured at 495 nm vs. the pH values. **(E)** Second derivative of the absorbance as a function of pH indicating pKa values.

On the other hand, the product 3aa was evaluated at different pH values (2 < pH < 12). A gradual colour change was observed as the pH of the medium increased. The colour evolved from a light red at pH values between 2.0 and 4.0, passed through a yellow at pH 5-9, and acquired a deep orange colour at pH 10-12 (Figure 2A). The product remained dissolved in the different aqueous solutions, except for concentrations above 1.5 × 10^–4^ mol L^−1^, where at pH values between 6 and 8 a suspended solid was observed after 30 min. The colour changes of 3aa corresponded to transitions of absorbance peaks between 380 and 550 nm. As it can be seen in Figure 2B, the spectra at pH 2-4 showed a band around 390 nm. As the pH increased from 5 to 9, a shift to longer wavelengths (∼450 nm) was observed. Then, from pH 10 to 12 there was a shift again towards longer wavelengths, giving a band around 490 nm. In addition, the UV-Vis spectra of the starting substrates that gave rise to **3aa** revealed bands only below 400 nm (see Supplementary Figure S41), showing that the bands for **3aa** in the visible region corresponded exclusively to the product.

Besides, the reversibility of the acid-base balance of **3aa** was also assessed by UV-Vis spectroscopy after the addition of HCl or NaOH .1 M solutions. Balance was considered to be reversible when the spectrum recorded for the compound at a given pH value, after considerably changing the acidity or basicity conditions, recovered the initial form if the pH returned to the original value. To compare the spectrum recorded at the beginning of the experiment with the ones obtained at the same pH, but after deliberate pH variations, the similarity coefficient was employed (Garrido et al., 2004), that is the cosine of the angle between the vectors representing the two spectra to be compared. The more similar the spectra are, the closer the cosine value will be to 1. Figure 2C shows the similarity coefficients obtained for three pH values: 3.0, 7.0 and 11.0, compared for two cycles of pH variation. As it can be seen, the coefficients obtained were higher than .995 in all cases, confirming the reversibility of the **3aa** product at different pH values.

Reversibility study was also performed for THIQ **2a** as a reference (see Supplementary Figure S42). It was observed that THIQ showed a band centered at 287 nm in MeOH and that after the addition of HCl, the spectrum showed no change. However, when NaOH was added, it was observed that the intensity of the band at 287 nm decreased markedly, and a band appeared at 348 nm. This behaviour would make sense considering the deprotonation of the hydroxyl groups. It is important to note that the spectra were reversible either from basic to acidic media or *vice versa*.

The spectra recorded for the aqueous solutions of **3aa** at different pH values suggested the presence of several species involved in an acid-base balance. To assess the pK_a_ values, a spectrophotometric study was performed. The results evidenced the presence of two isosbestic points around 402 nm and 462 nm. Figure 2D shows the chart corresponding to the absorbance measured at 495 nm vs. the pH values. The turning points in the graph are related to the pK_a_ values. A more precise identification of the pKa may be obtained using the second derivative of the absorbance as a function of pH (Figure 2E). The values were pK_a1_: 4.4 and pK_a2_: 9.5. These pK_a_ would correspond, in principle, to the deprotonation of the OH groups of the catechols. Although phenols or catechols are known to have pK_a_ around 9-13, when these groups are conjugated, deprotonation of these groups can occur at pH values of 4 (Dangles, Fenger, 2018).

### 3.5 Study of product 3aa by ^13^C NMR

**FIGURE 3:**
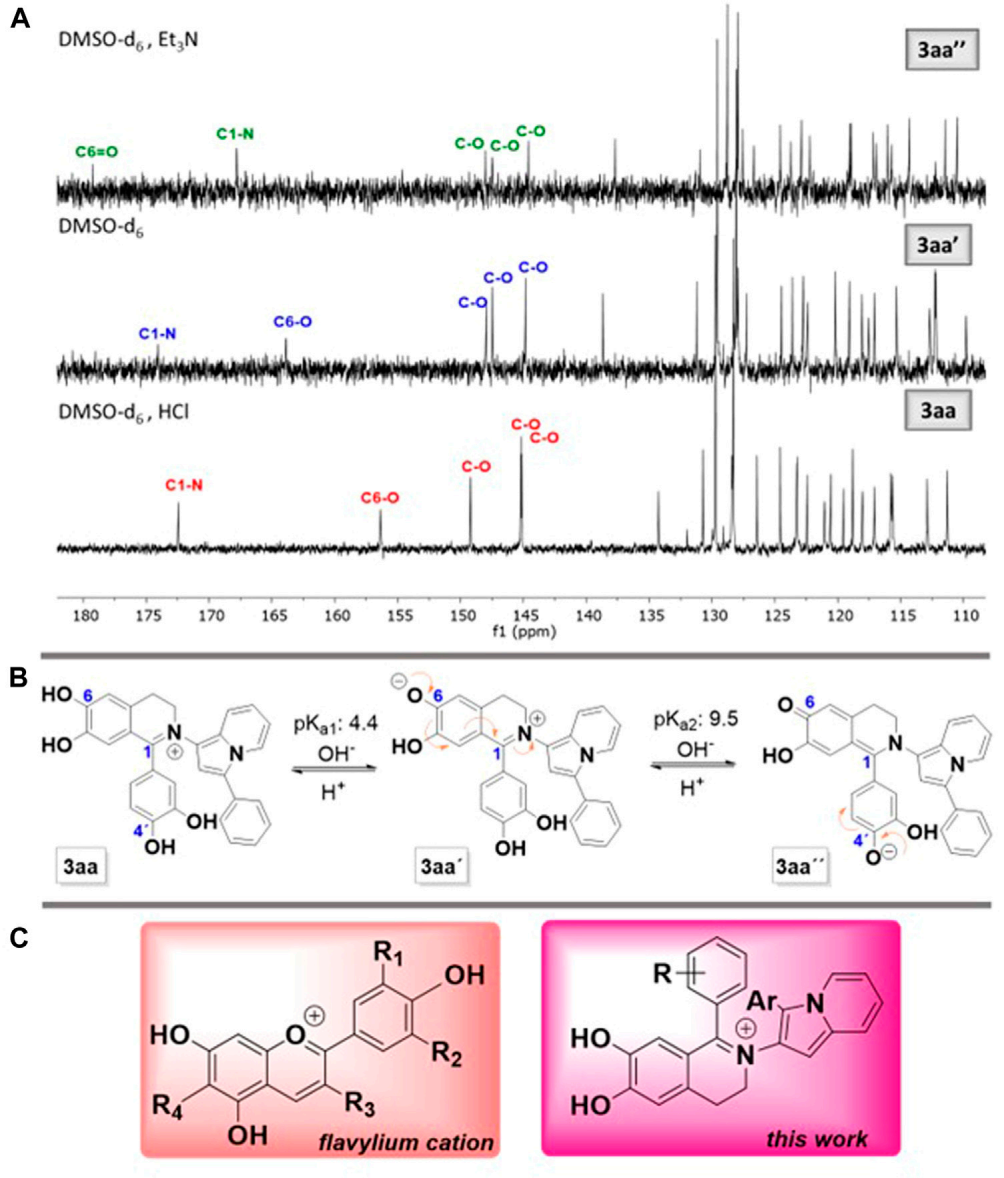
**(A)** ^13^C NMR spectra of product **3aa** in different media with a proposed assignment of C6. **(B)** Proposed structures at different pHs. **(C)** Comparison between the structure of the flavylium cation and indolizines **3**.

To obtain structural information about the possible species that predominated at different pH values, we decided to carry out a study by ^13^C NMR. It consisted in analysing the spectra of the sample dissolved in DMSO-d_6_, DMSO-d_6_/HCl and DMSO-d_6_/Et_3_N (Figure 3A). The spectra of **3aa** in DMSO-d_6_ and in DMSO-d_6_/HCl in the zone above 140 ppm, which correspond mainly to those C attached to the OH and C=N^+^, showed the presence of the iminium bond and therefore the absence of the signal at 58 ppm corresponding to the aliphatic C1 in the starting THIQ **2a** (see Supplementary Figures S2, S3 **)**. Furthermore, it was observed that C6 shifted from 156 ppm in acidic medium (DMSO-d_6_/HCl) to 164 ppm in DMSO-d_6_ medium. The assignment of C6 represents a proposal based on the ^13^C NMR spectra of the product 3aa and its behaviour in different media (for this analysis see Section 5 in Supplementary Material S1). With this information in hand, we proposed that this change could be related to the deprotonation of hydroxyl group located at C6 of the THIQ ring giving structure **3aa’** (Figure 3B).

On the other hand, the spectrum in DMSO-d_6_/Et_3_N (Figure 3A, top) showed a signal around 180 ppm, which was assigned to C=O of quinone **3aa’’**. Finally, the samples analysed in the three media showed a red colouration in the three systems studied, possibly indicating that in all structures there was an extended conjugation.

The structures of products **3**, their colour and behaviour at different pH values, resemble that of the flavylium cation of anthocyanins (Figure 3C), which changes from red to blue colour depending on pH (Dangles, Fenger, 2018).

In the case of product **3ba**, as commented above, we obtained the product as a mixture of two related structures, visualized as two red spots by TLC analysis. These products were isolated with yields of 30% and 16% for the most (FMR) and least (FLR) retained fraction in the TLC respectively. It was observed that the FMR corresponded to indolizine **3ba**, showing the same ^13^C NMR spectra in both DMSO-d_6_ and DMSO-d_6_/HCl (see Supplementary Figure S44). This fact possibly indicated that we were already in the presence of the more acidic species, thus showing no changes in the NMR signals after the addition of HCl. The FLR however, showed different spectra in the presence of DMSO-d_6_ and after the addition of hydrochloric acid (see Supplementary Figure S45). We speculated that the FLR fraction could correspond to a more basic species that, could be protonated after the addition of acid. This was also supported by the fact that, after acidic treatment, the FLR spectrum showed to be quite similar to the FMR spectrum, both fractions thus seeming to converge to the same species in acidic medium.

## 4 Conclusion

In summary, we have reported the synthesis of new indolizines dyes bearing a catechol moiety with varied substitution pattern. These products were obtained through a three component one-pot reaction between pyridine-2-carbaldehyde, an alkyne and a THIQ promoted by CuNPS/C. The reaction proved successful for aromatic alkynes such as phenylacetylene, 4-methylphenylacetylene and 4-methoxyphenylacetylene, giving yields between of 31%–34%. However, it failed when electron-deficient aromatic alkynes or aliphatic ones, were employed. The scope of the reaction was also studied by using different THIQs as starting amines. It was observed that the reaction took place when the C1 of THIQ had mainly an aryl group as a substituent. On the other hand, when the reaction was carried out with a substrate without the OH groups on the C6 and/or C7 of the THIQ ring, it turned bright red within a few hours, but a complex mixture of coloured byproducts was observed by TLC.

Regarding the possible mechanism for this reaction and based on previous reported works, it was proposed the formation of a propargylamine followed by oxidation of THIQ ring to produce an iminium bond. Finally, a copper-promoted cycloisomerization of the resulting intermediate, could take place, giving the final products **3**. Based on our experimental observations and previous studies reported by other authors, we postulated a redox catalytic process promoted by CuO and Cu_2_O species on the surface of the CuNPs/C catalyst.

Concerning the study of the optical properties of these new dyes, low fluorescence of **3aa** in DMSO and MeCN was detected, whereas no fluorescence was observed in MeOH or AcOEt. UV-Vis analysis of product **3aa** was evaluated at different pH values (2 < pH < 12). The colour evolved from a light red to yellowish and finally to a deep orange colour, when analysed from acid to basic pH values. Under the same study of **3aa**, the presence of two isosbestic points at pK_a1_: 4.4 and pK_a2_: 9.5 was unveiled. This led us to propose the existence of three different structures depending on the pH, arising from the first and second deprotonation of the OH groups of the THIQ ring of **3aa**. On the other hand, product **3aa** was proved to be reversible by UV-Vis spectroscopy, after acid-base treatment.

Finally, ^13^C NMR study at different pH values, confirmed what was observed in UV-Vis, allowing us to assign the first deprotonation (pK_a1_: 4.4) to the hydroxyl group located in C6 and the second one (pK_a2_: 9.5) to the OH in C4, giving a quinone.

## Data Availability

The original contributions presented in the study are included in the article/Supplementary Material, further inquiries can be directed to the corresponding author.
