# Supplementary material for: Novel pH-sensitive catechol dyes synthesised by a three component one-pot reaction
Source: Front Chem. 2023 Jan 10;10:1116887. doi: 10.3389/fchem.2022.1116887 (PMC9871305; doi:10.3389/fchem.2022.1116887)
Supplement: Supplementary file 1 [file DataSheet1.PDF]

Supplementary Material

Novel pH-sensitive catechol dyes synthesised by a three component one-pot reaction

Juan José Calmels, Leandro Aguilar, Juan Mancebo-Aracil, Gabriel Radivoy, Claudia Domini, Mariano Garrido, Miguel D. Sánchez, Fabiana Nador*

*** Correspondence:** Corresponding Author: fabiana.nador@uns.edu.ar

1 Spectra of THIQs

1.1 THIQ 2a

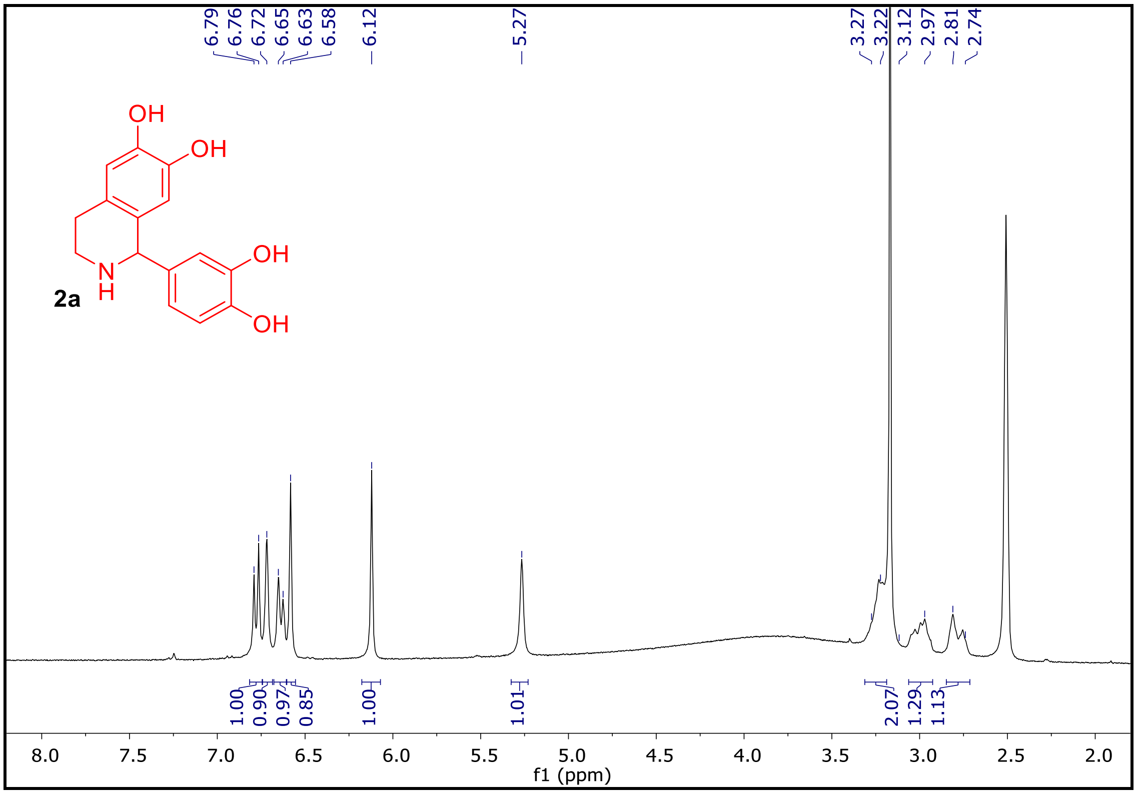

Figure S1. ^{1}H NMR of **2a** in DMSO-d_6

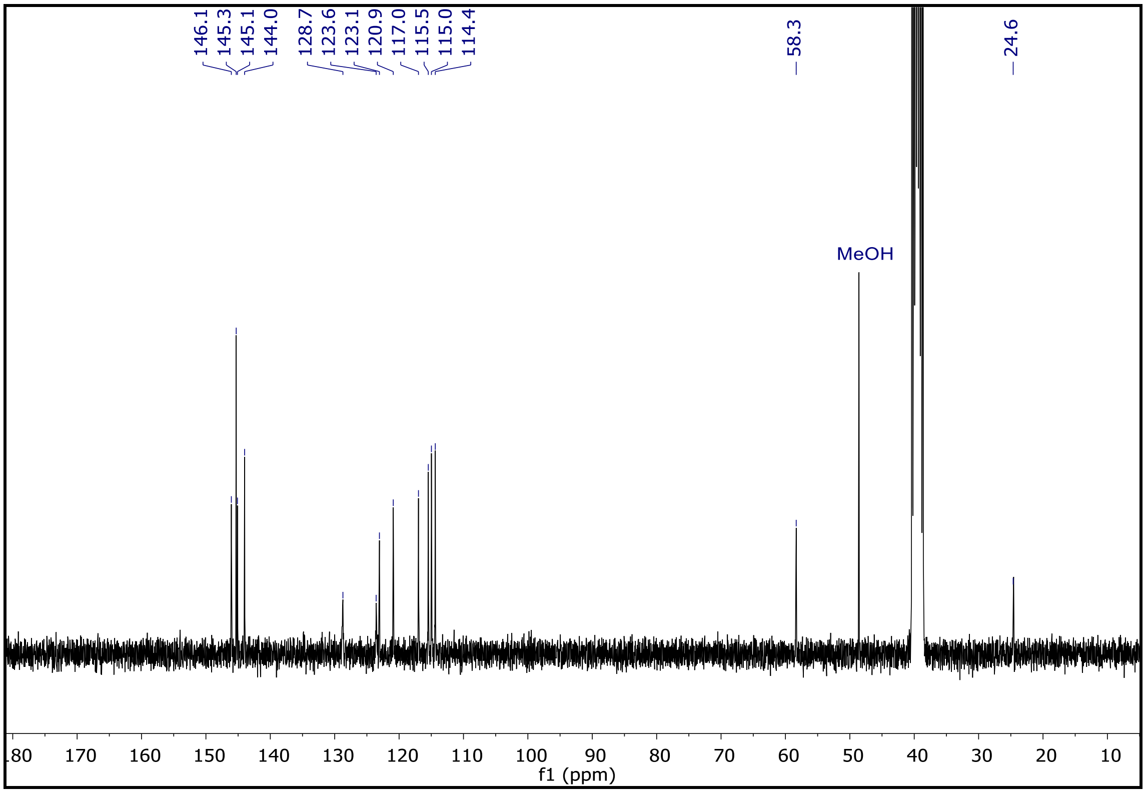

Figure S2. ^{13}C NMR of **2a** in DMSO-d_6

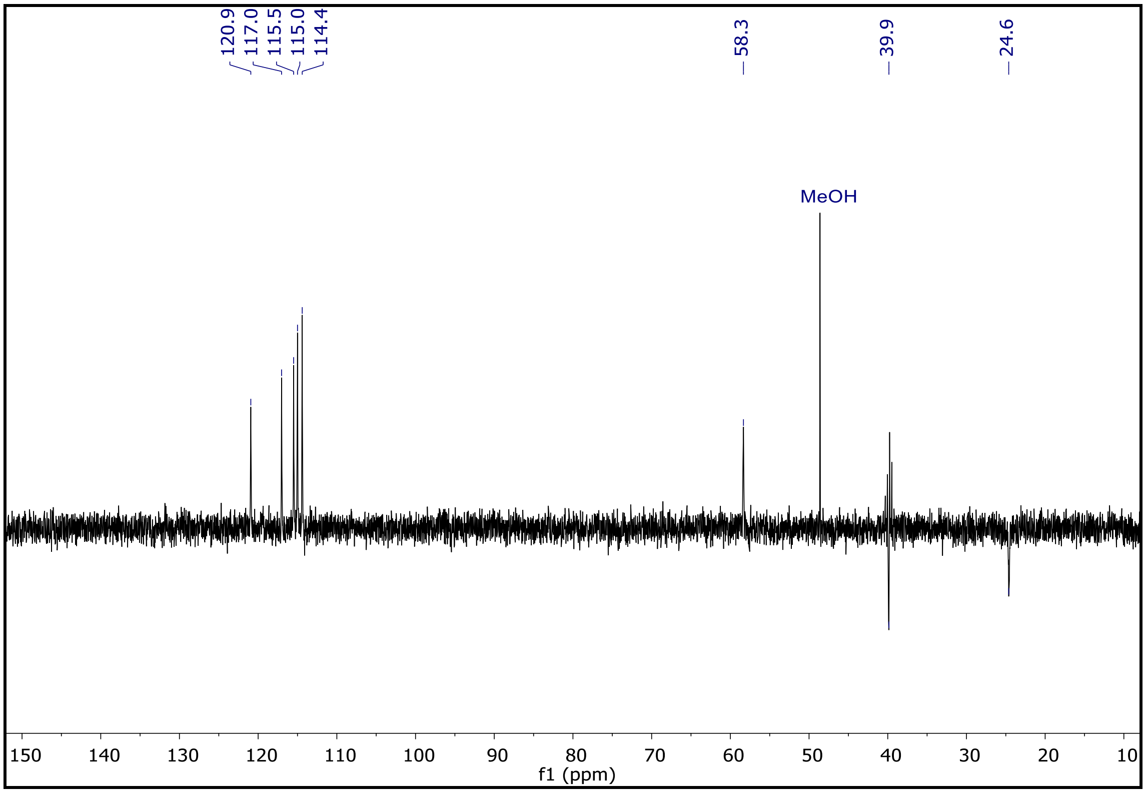

Figure S3. DEPT of **2a** in DMSO-d_6

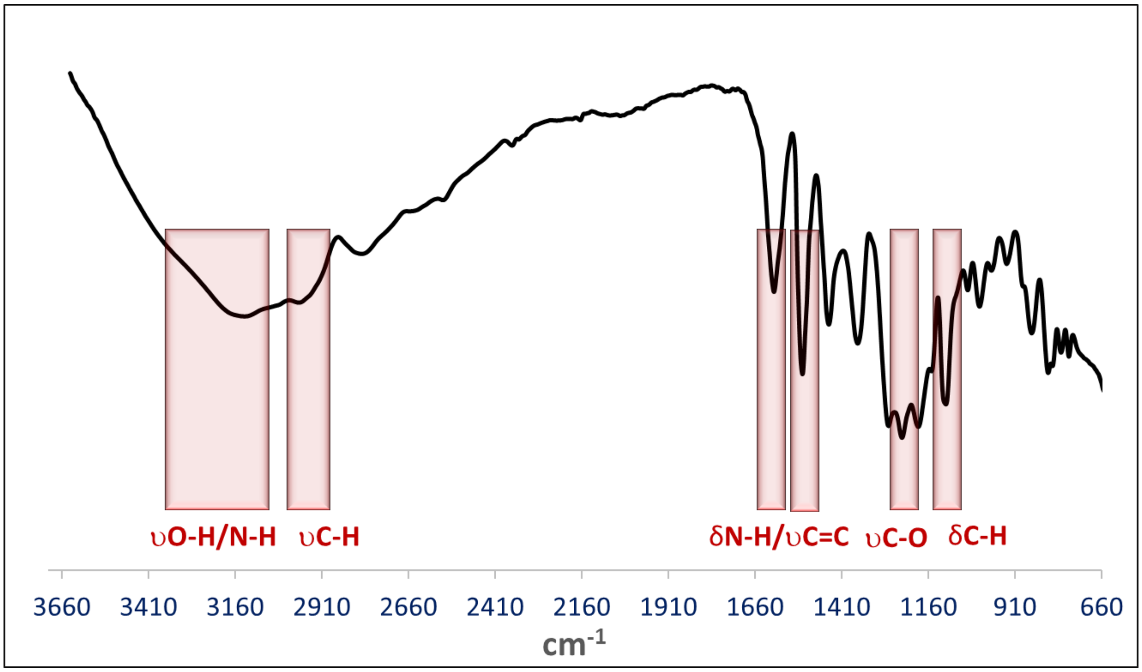

Figure S4. IR of **2a**

1.2 THIQ 2b

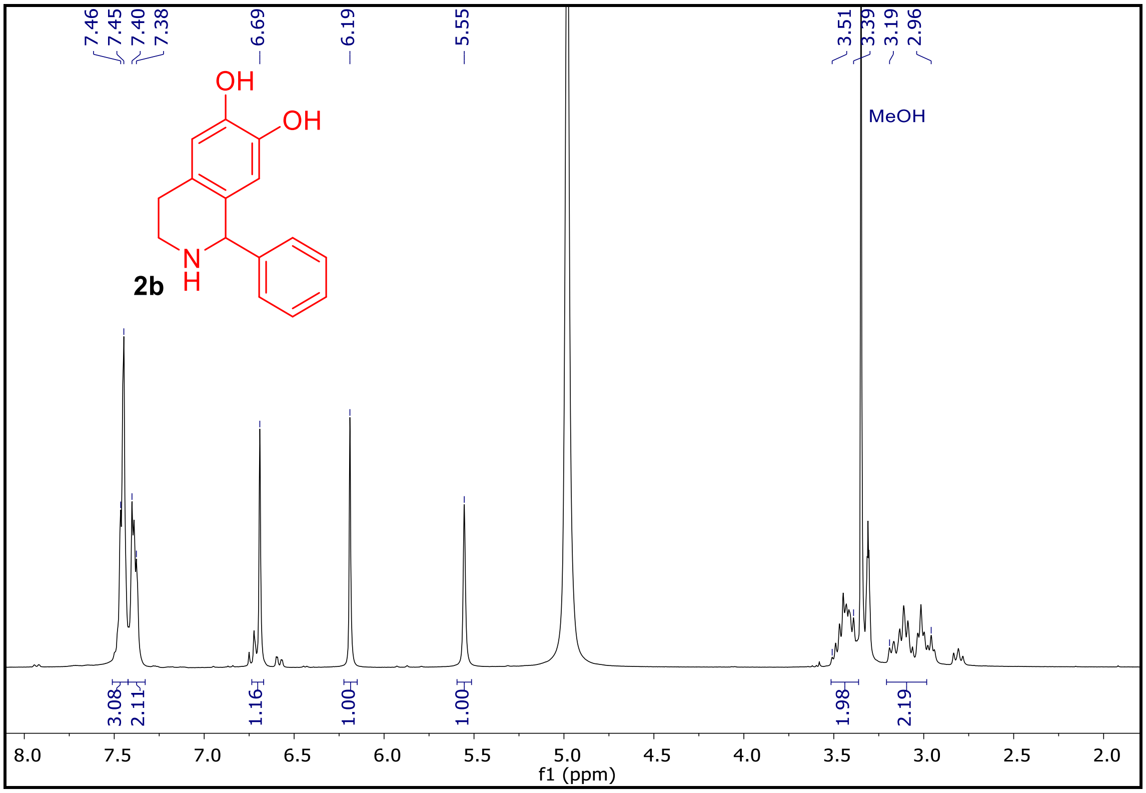

Figure S5. ^{1}H NMR of **2b** in MeOD-d4

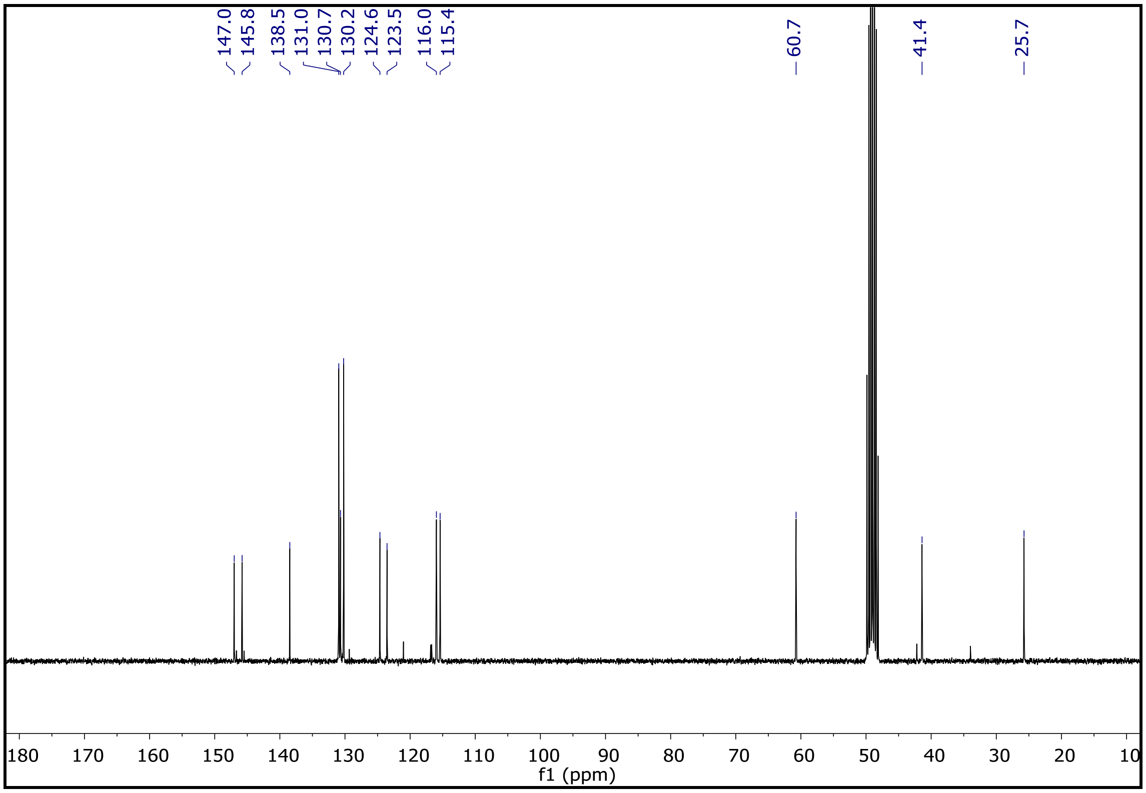

Figure S6. ^{13}C NMR of **2b** in MeOD-d$_4$

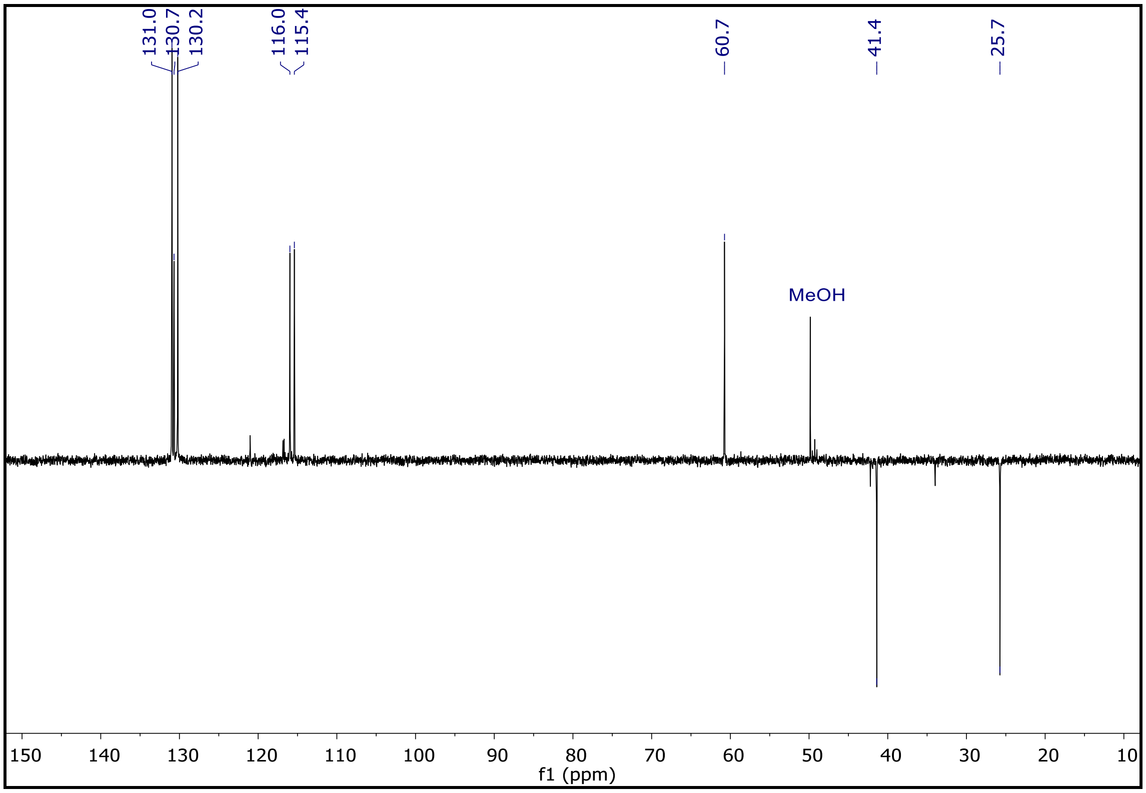

Figure S7. DEPT of **2b** in MeOD-d$_4$

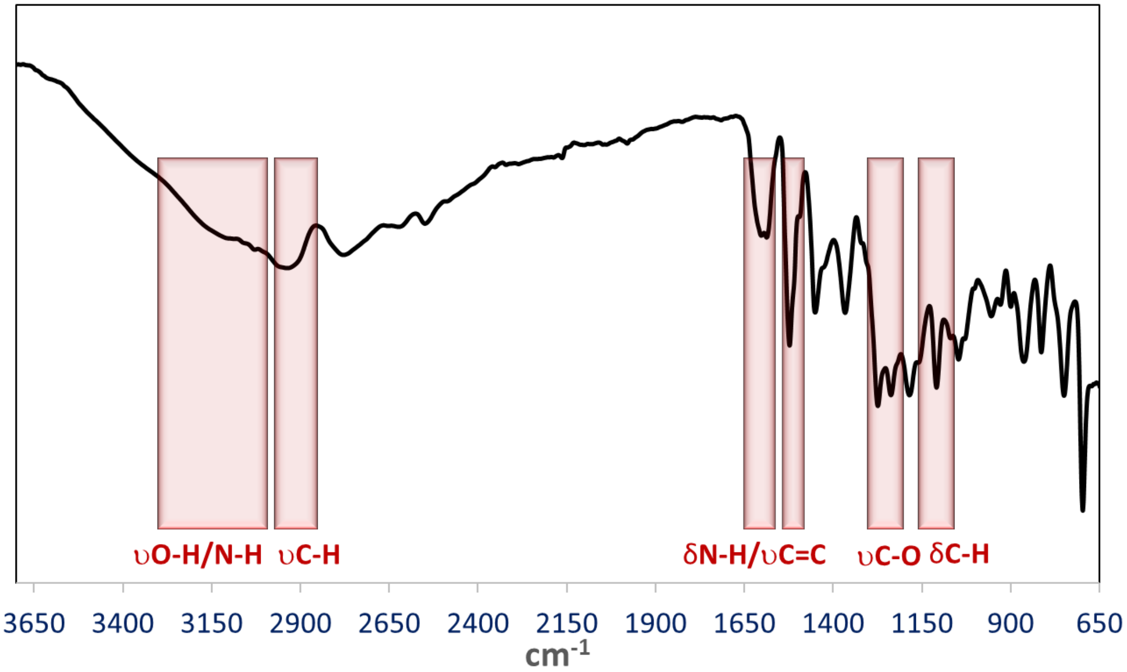

Figure S8. IR of **2b**

1.3 THIQ 2c

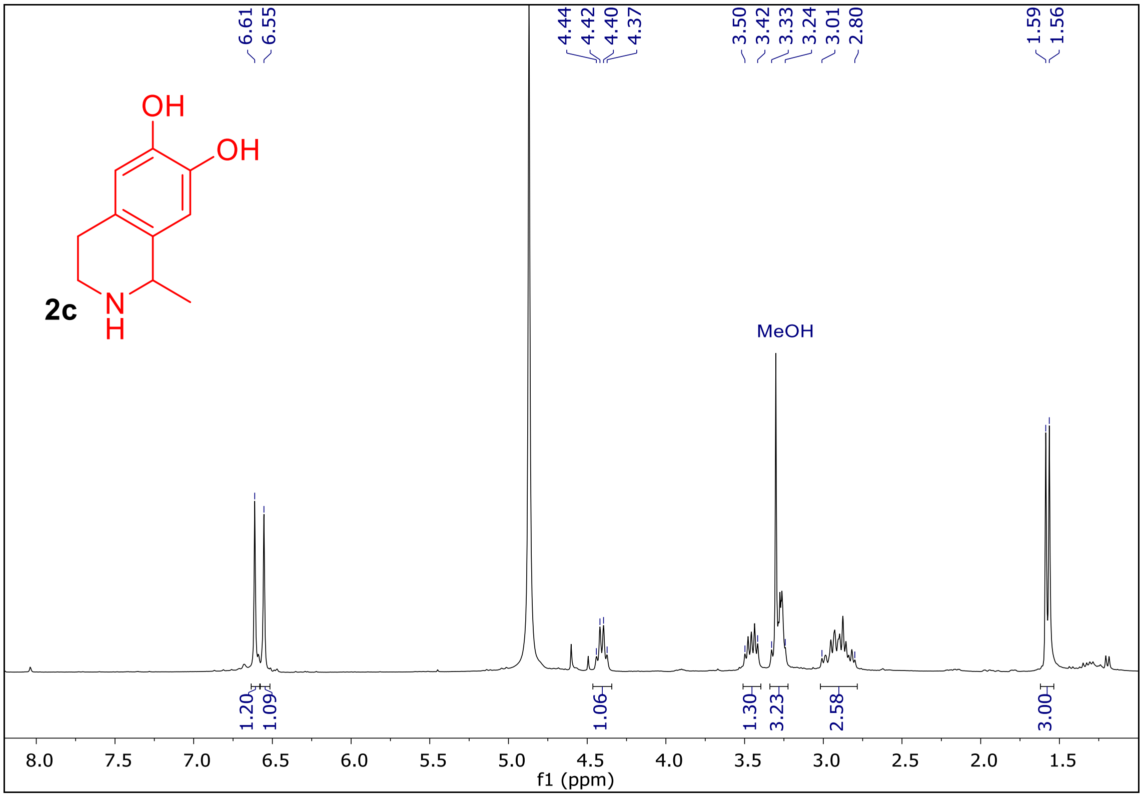

Figure S9. ^{1}H NMR of **2c** in MeOD-d_4

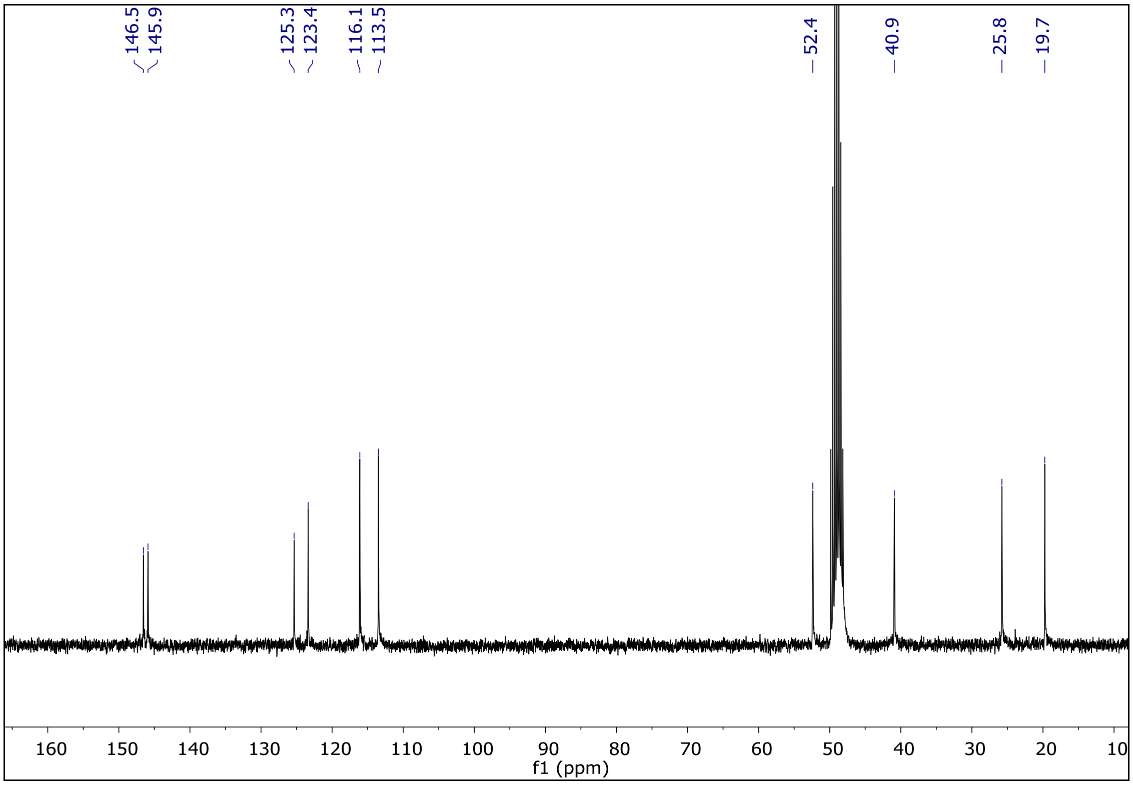

Figure S10. ^{13}C NMR of **2c** in MeOD-d_4

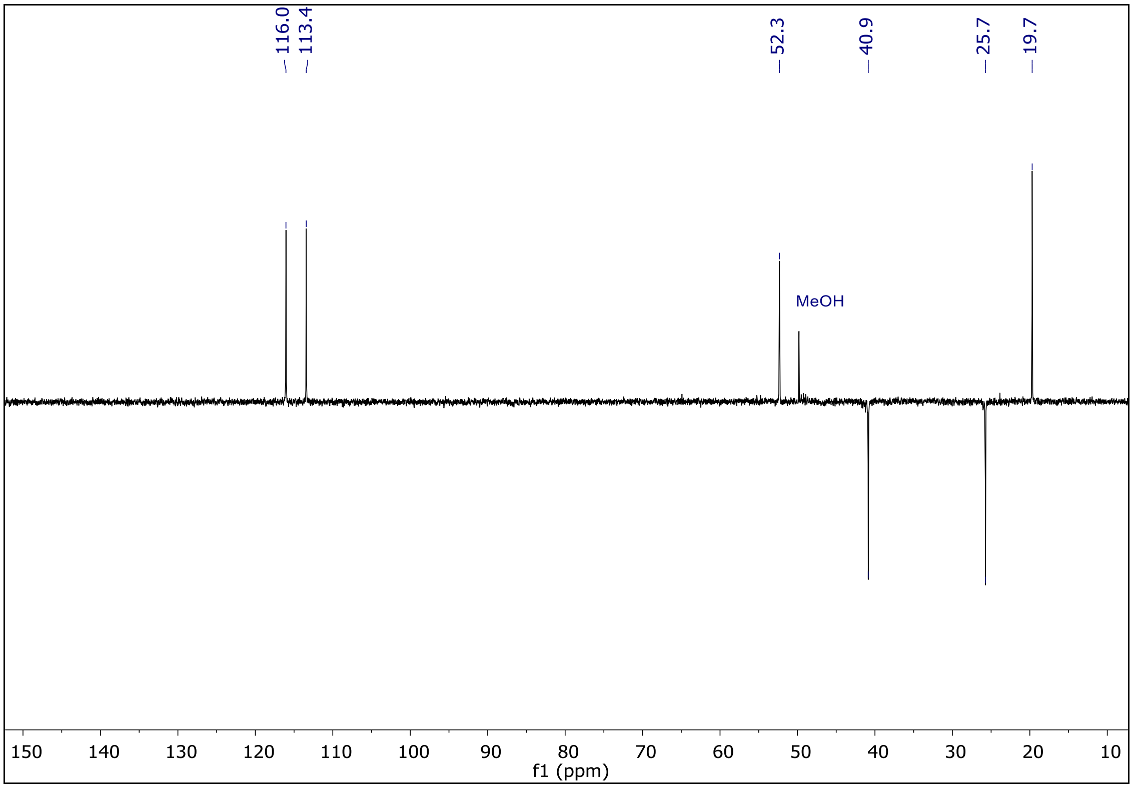

Figure S11. DEPT of **2c** in MeOD-d_4

1.4 THIQ 2d

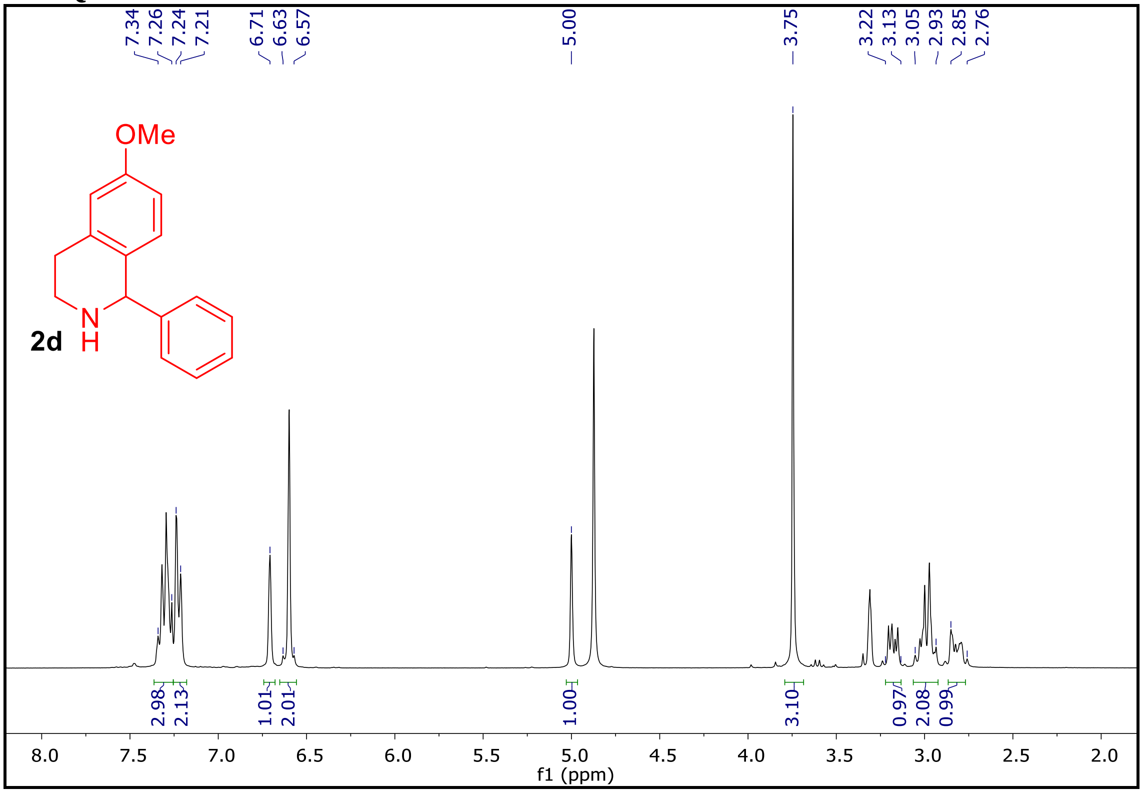

Figure S12. ^{1}H NMR of **2d** in MeOD-d_4

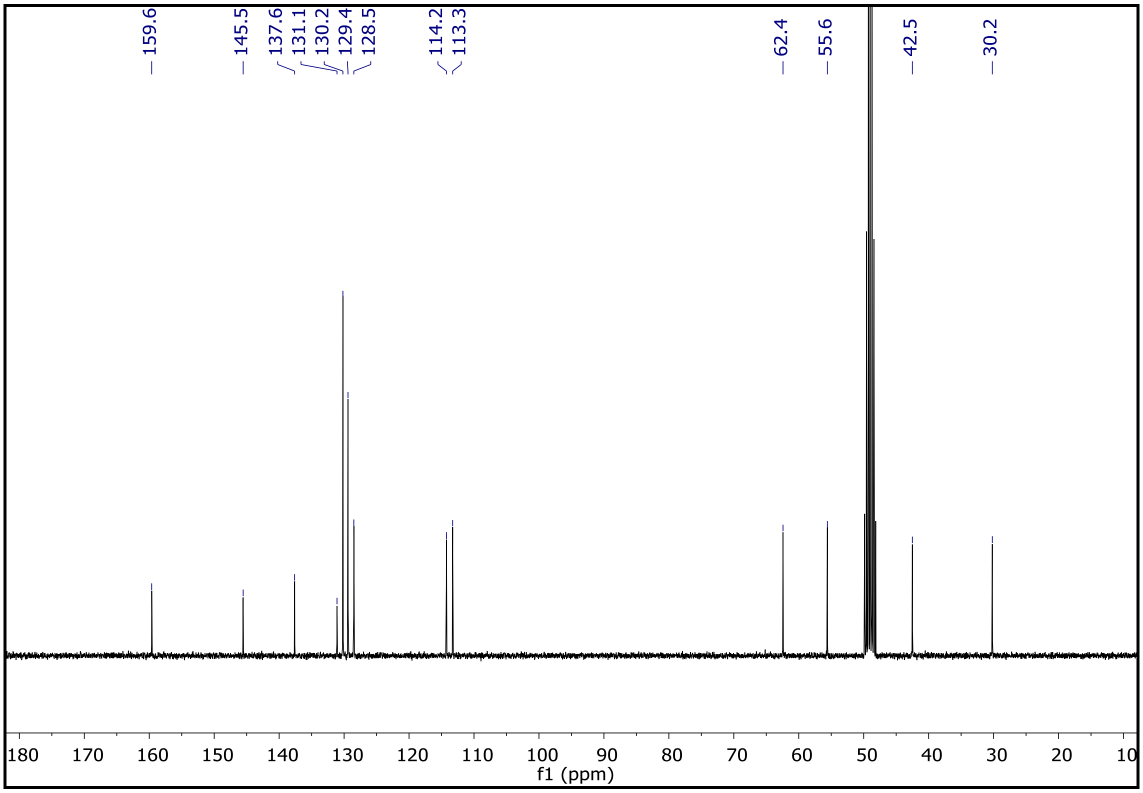

Figure S13. ^{13}C NMR of **2d** in MeOD-d_4

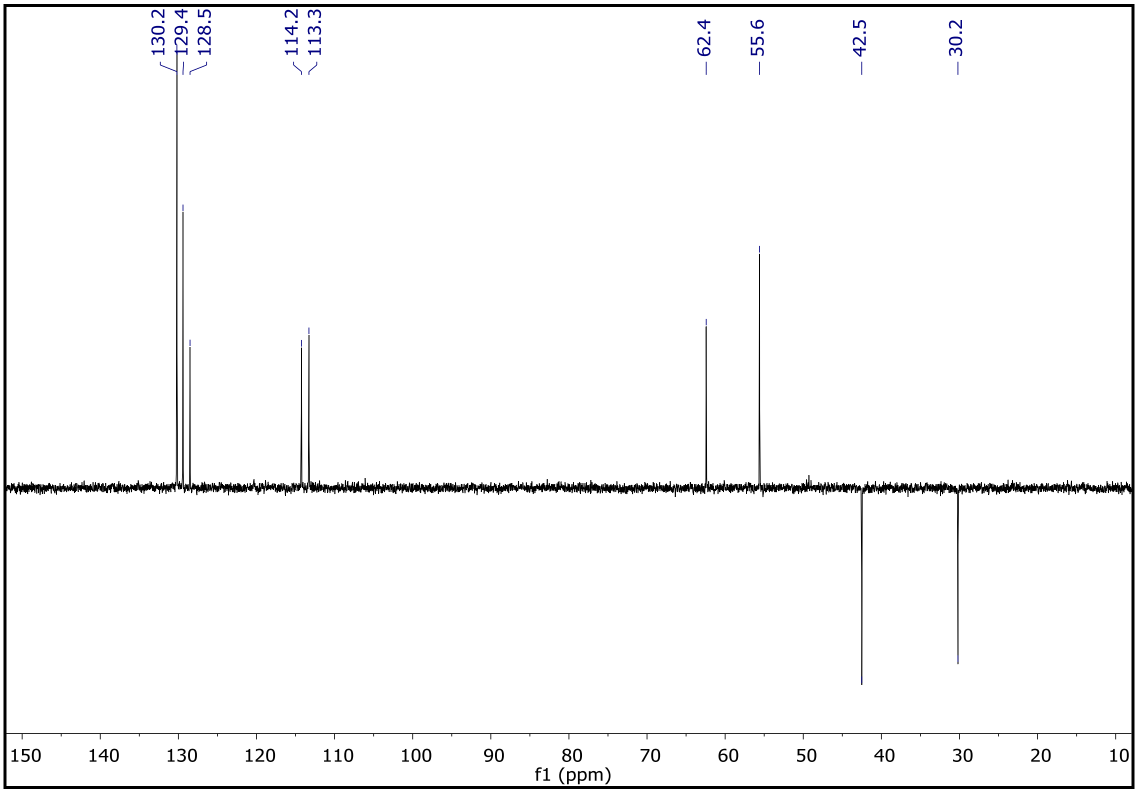

Figure S14. DEPT of **2d** in MeOD-d4

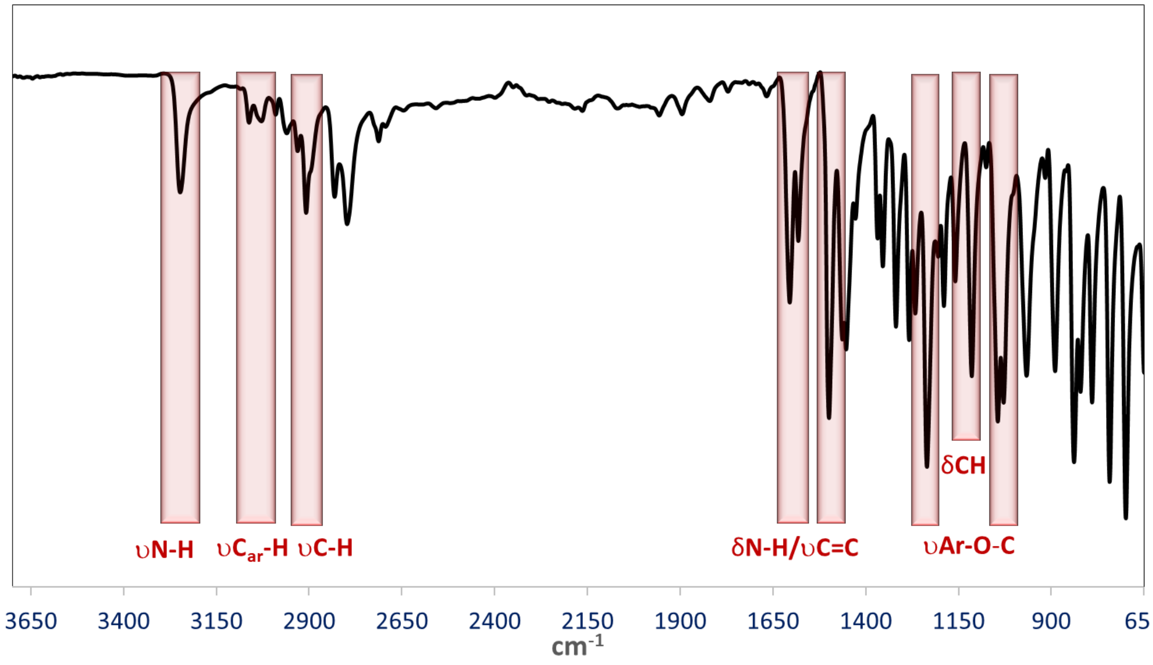

Figure S15. IR of **2d**

2 Spectra of products 3

2.1 Product 3aa

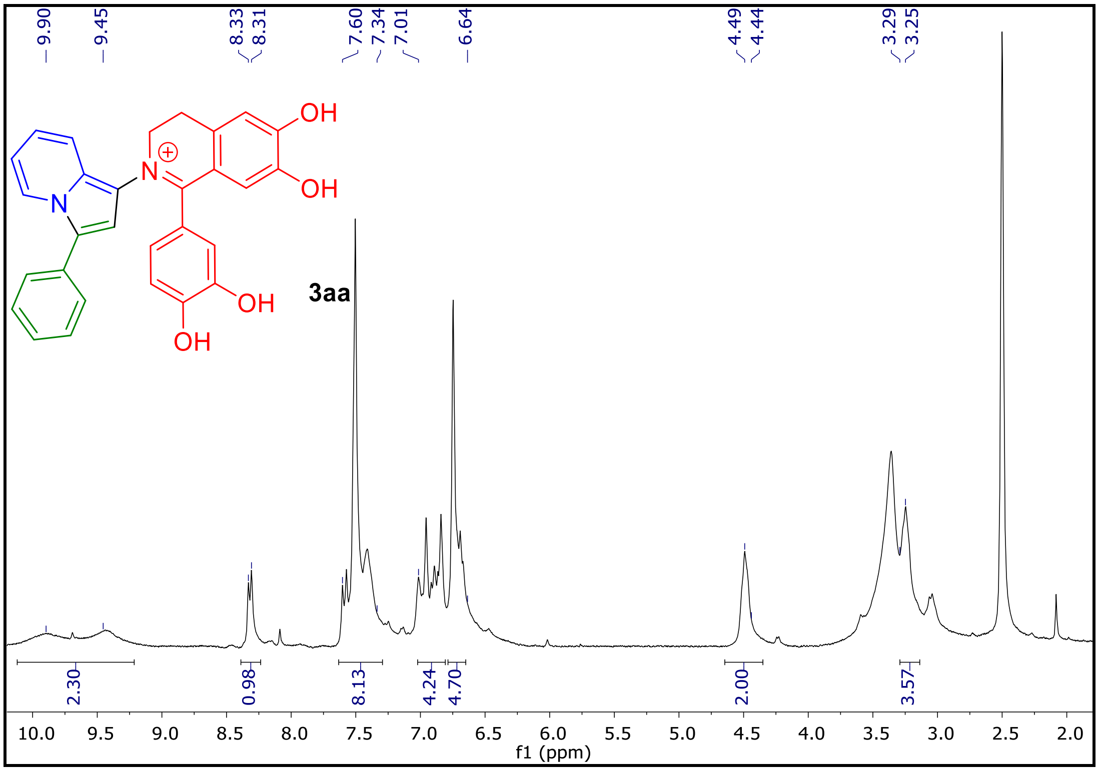

Figure S16. ^{1}H NMR of **3aa** in DMSO-d$_6$

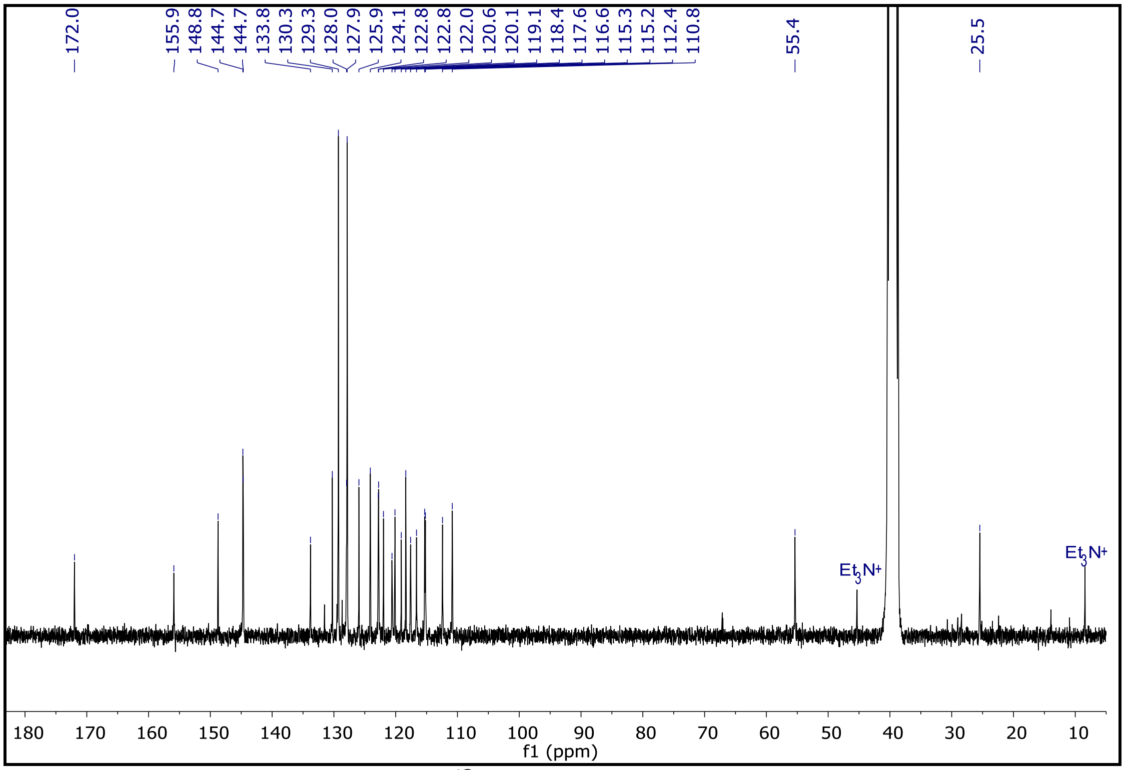

Figure S17. ^{13}C NMR of **3aa** in DMSO-d$_6$

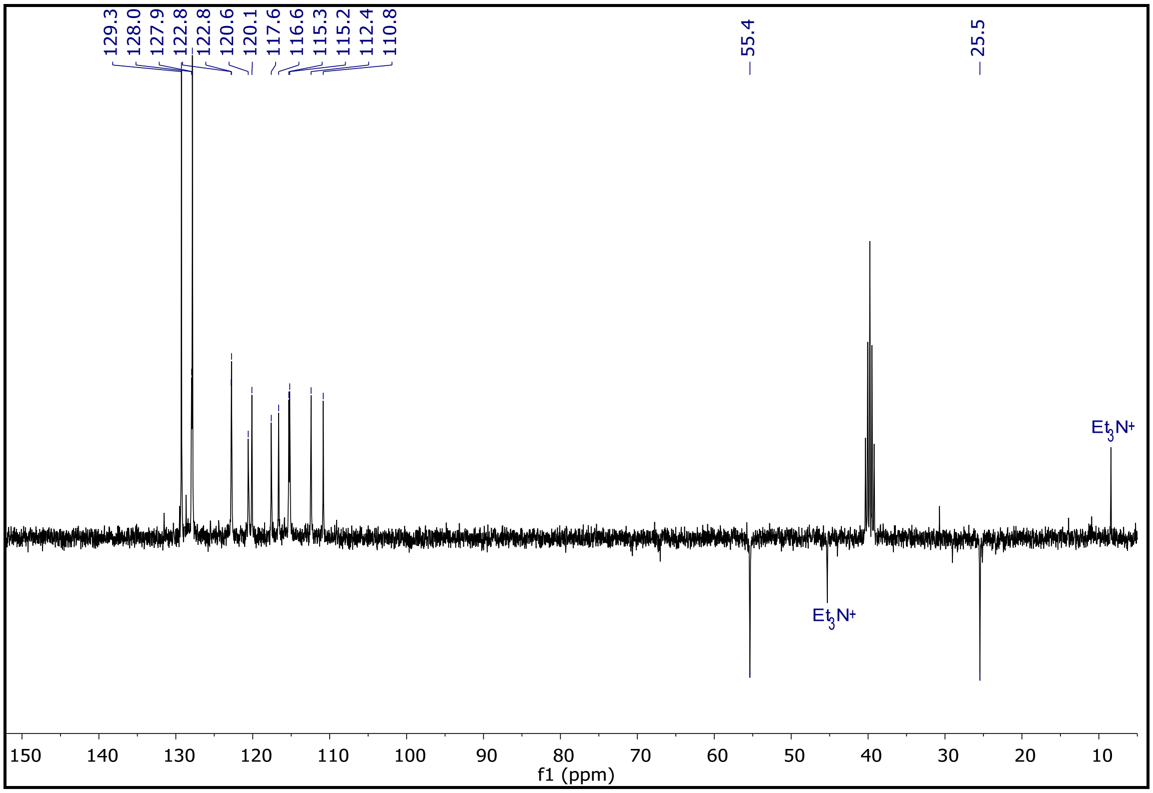

Figure S18. DEPT of **3aa** in DMSO-d_6

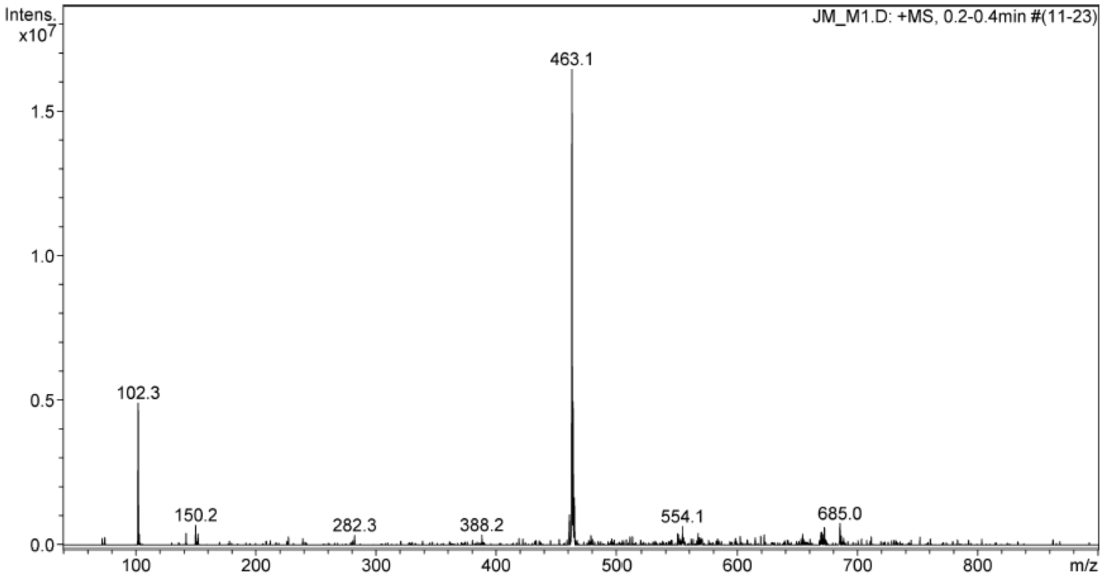

Figure S19. ESI-MS of **3aa**

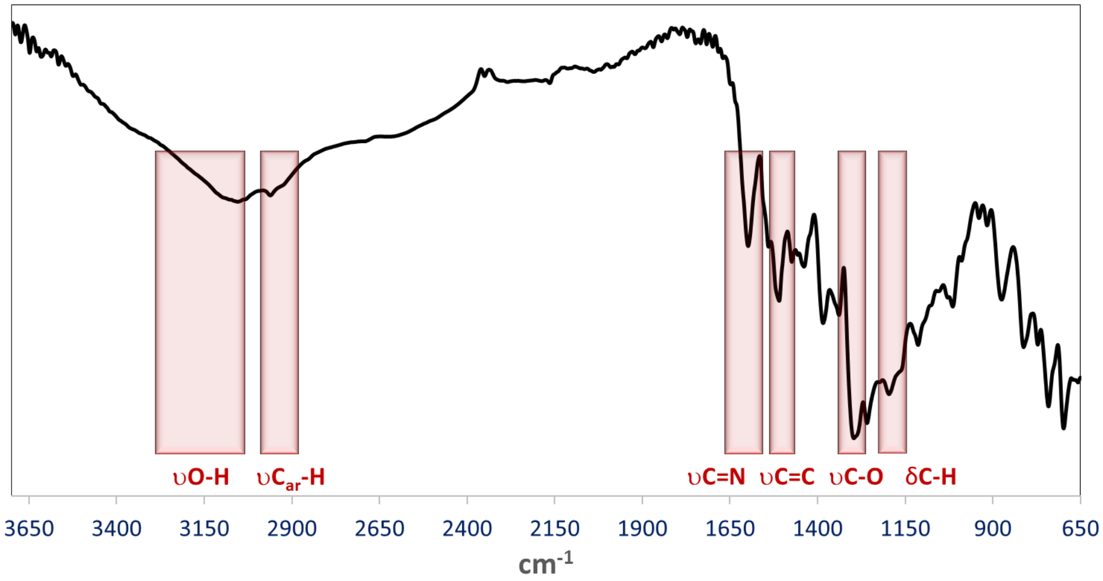

Figure S20. IR of **3aa**

2.2 Belstein and $AgNO_3$ test of product 3aa

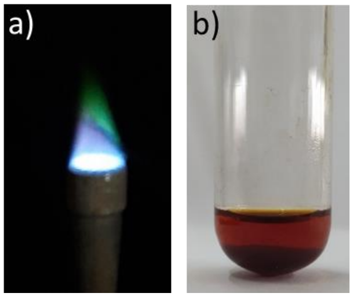

Figure S21. a) Positive Beilstein's test by a green flame caused by the formation of a copper halide. b) Positive $AgNO_3$ test by a precipitate of AgCl.

2.3 Product 3ab

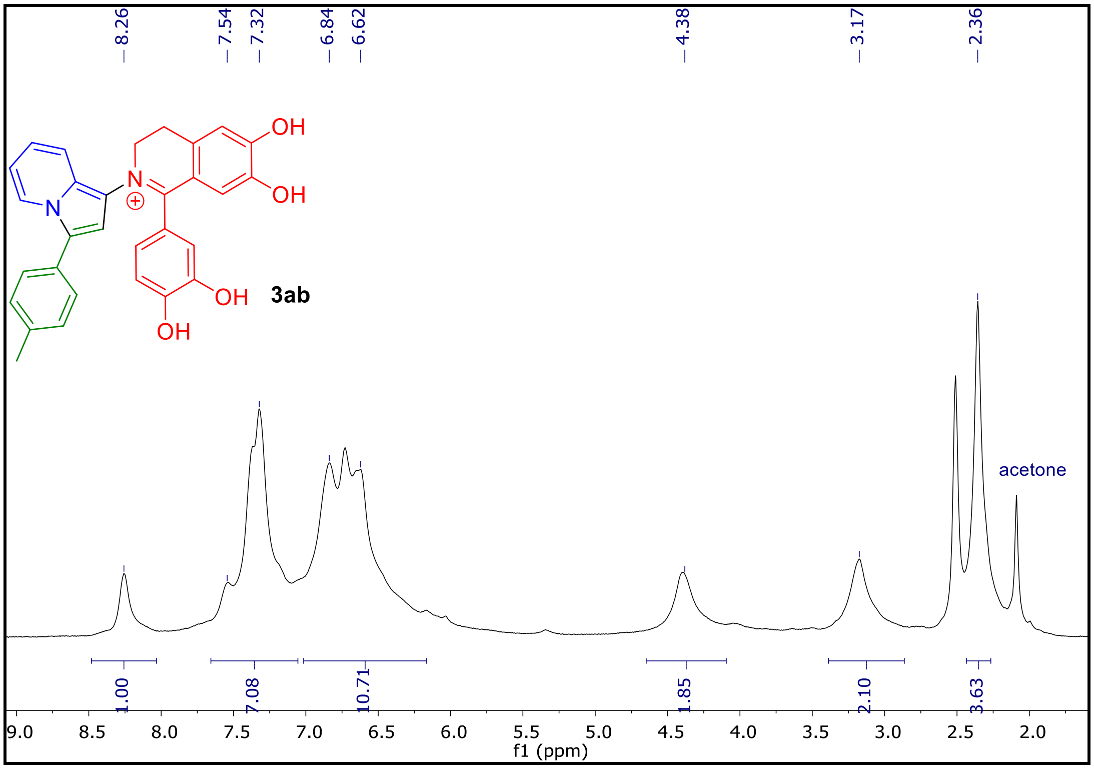

Figure S22. ^{1}H NMR of **3ab** in DMSO-d6

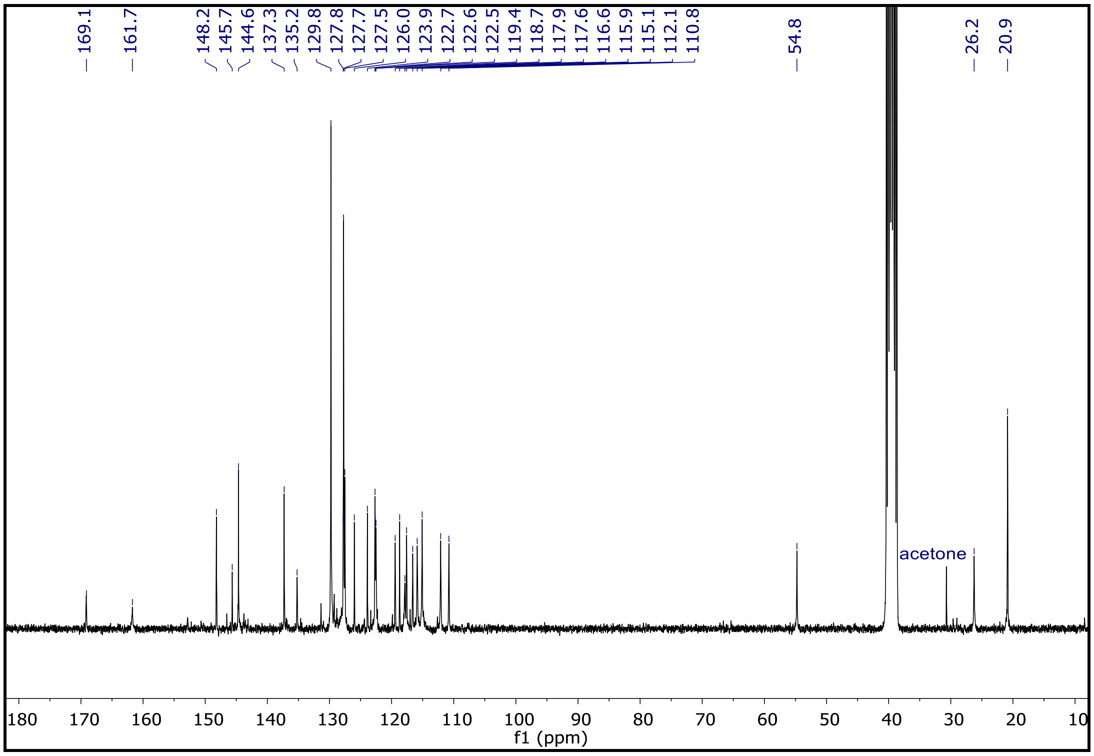

Figure S23. ^{13}C NMR of **3ab** in DMSO-d6

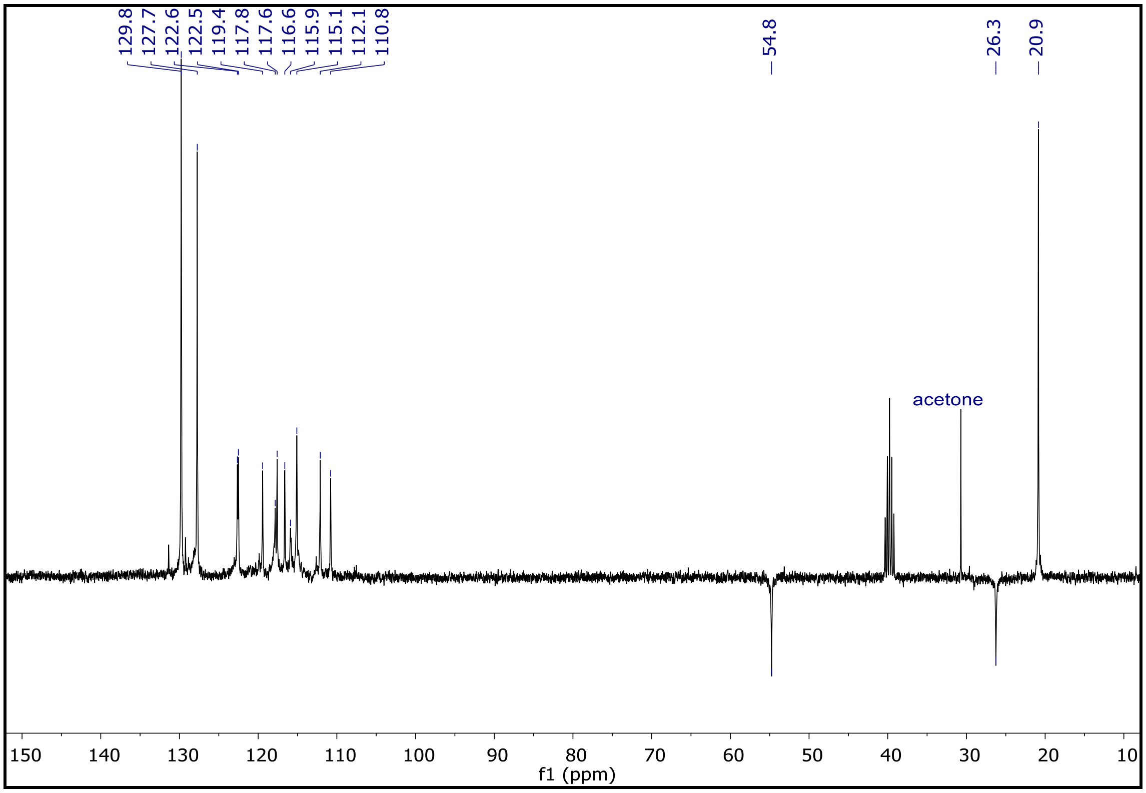

Figure S24. DEPT of **3ab** in DMSO-d$_6$

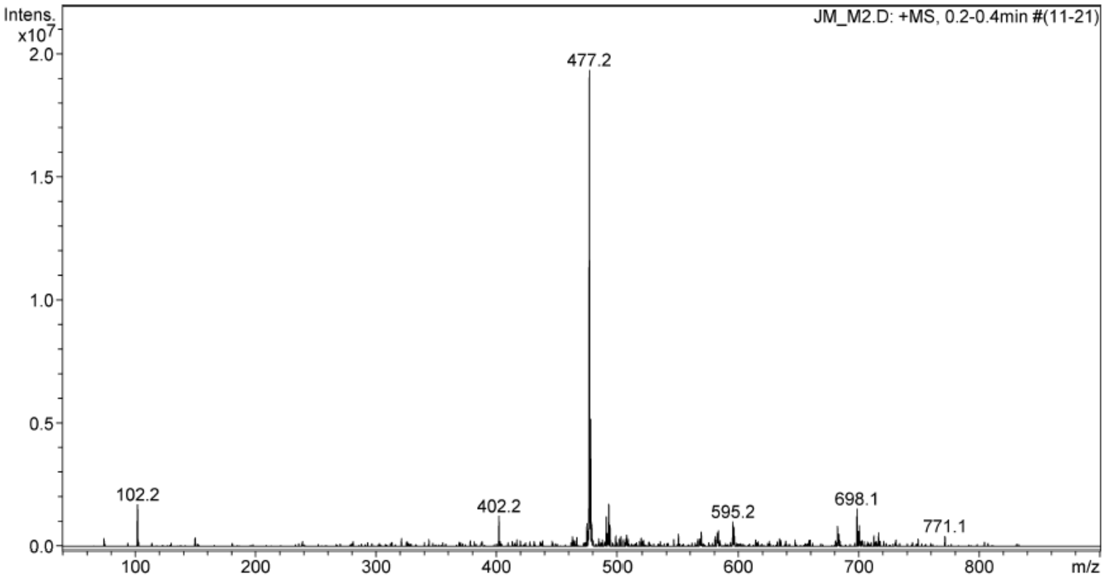

Figure S25. ESI-MS of **3ab**

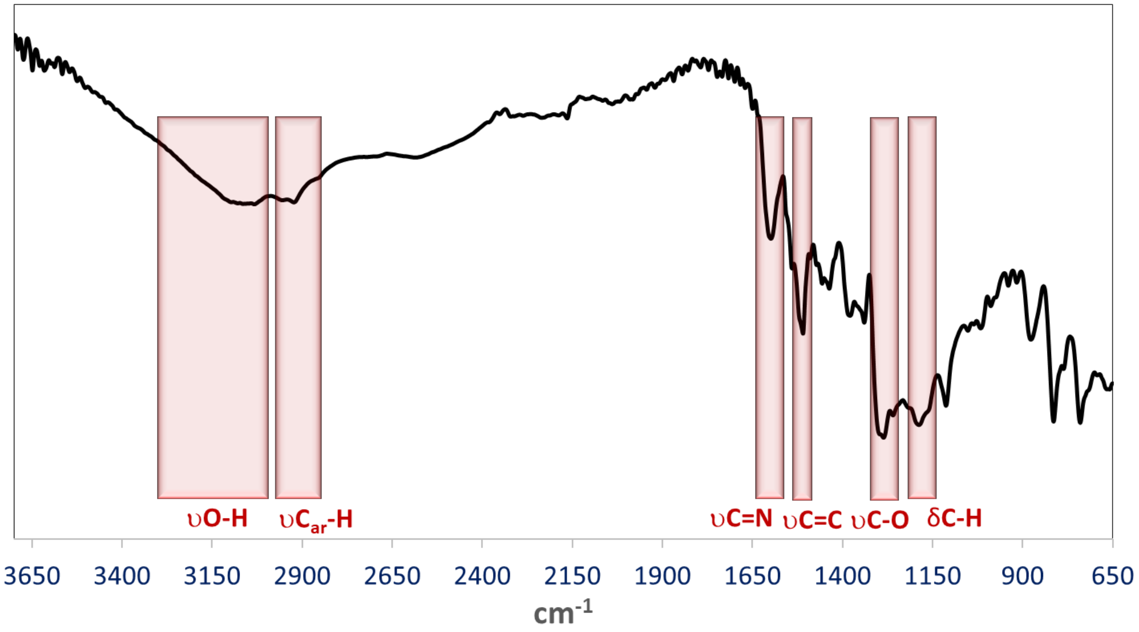

Figure S26. IR of **3ab**

2.4 Product 3ac

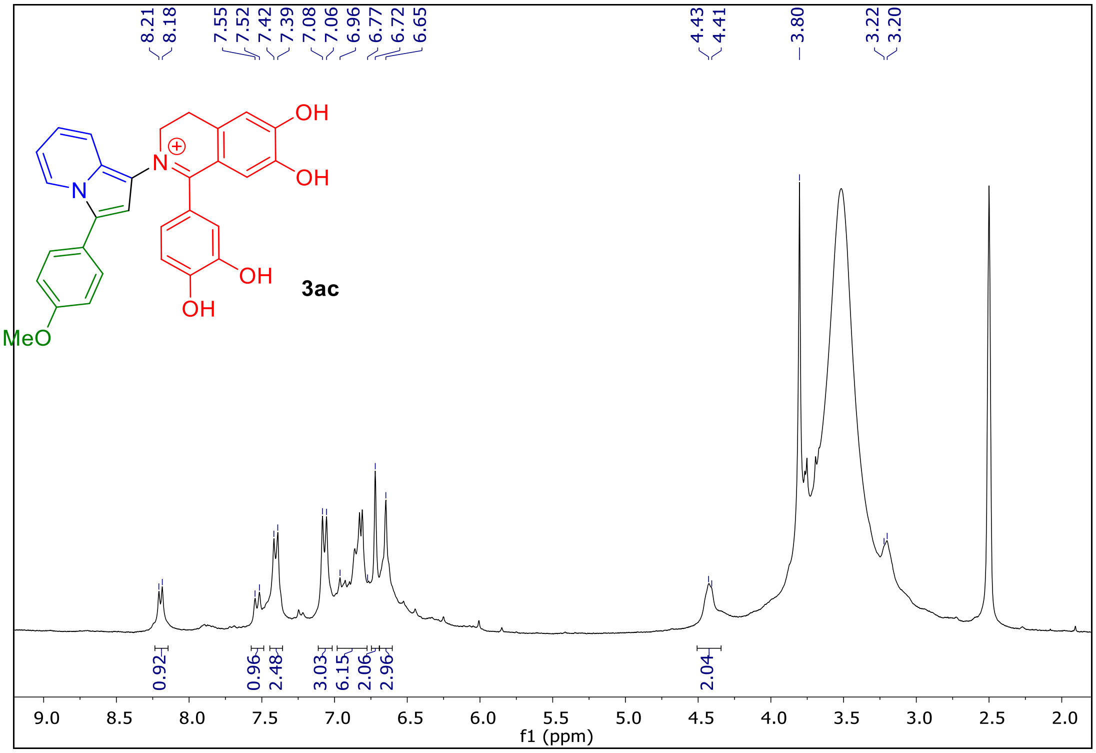

Figure S27. ^{1}H NMR of **3ac** in DMSO-d$_6$

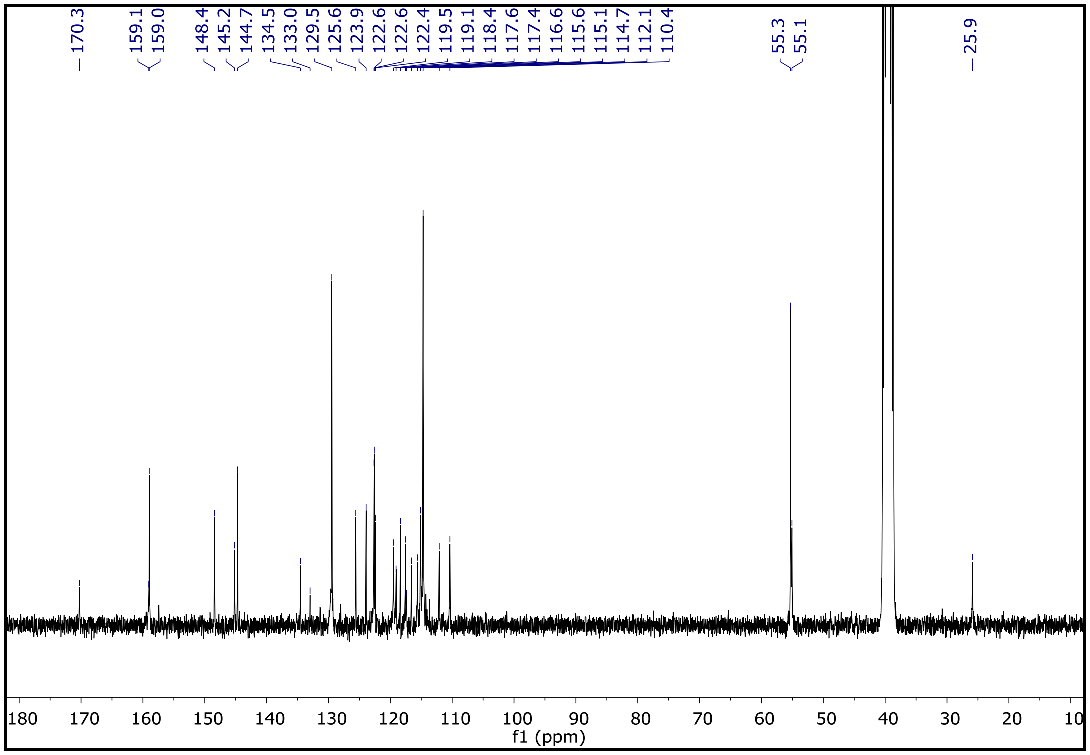

Figure S28. ^{13}C NMR of **3ac** in DMSO-d_6

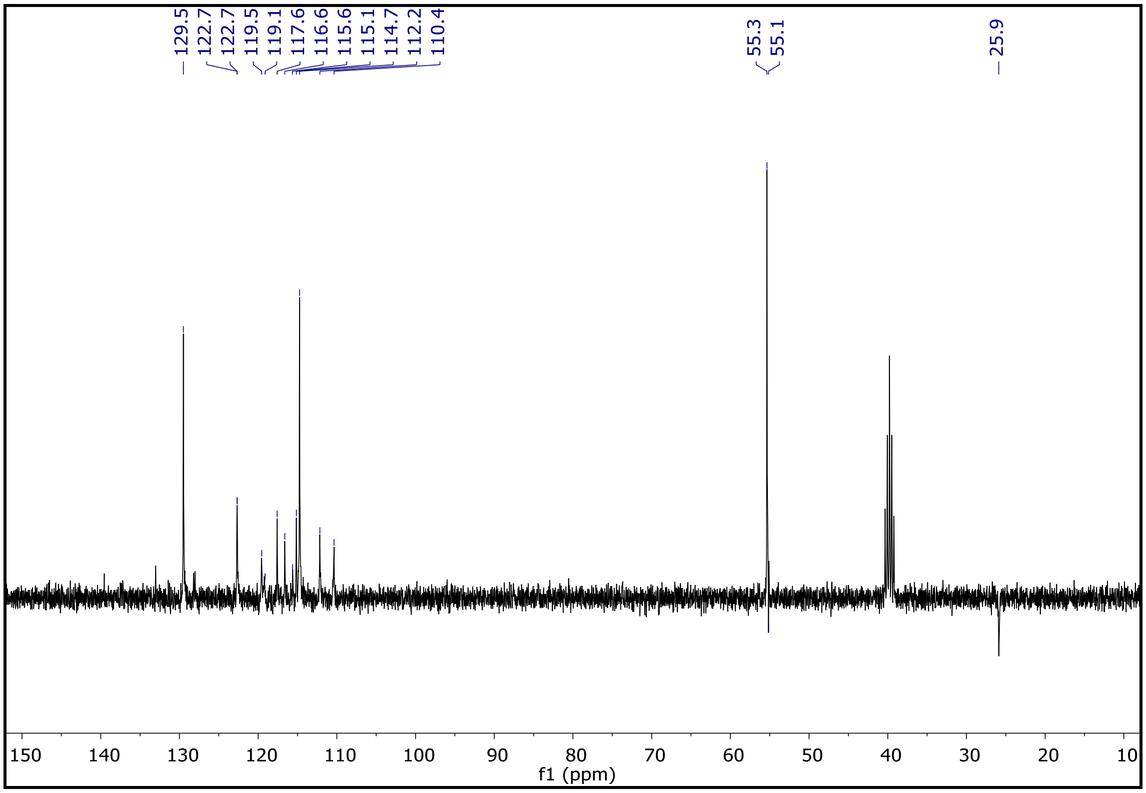

Figure S29. DEPT of **3ac** in DMSO-d_6

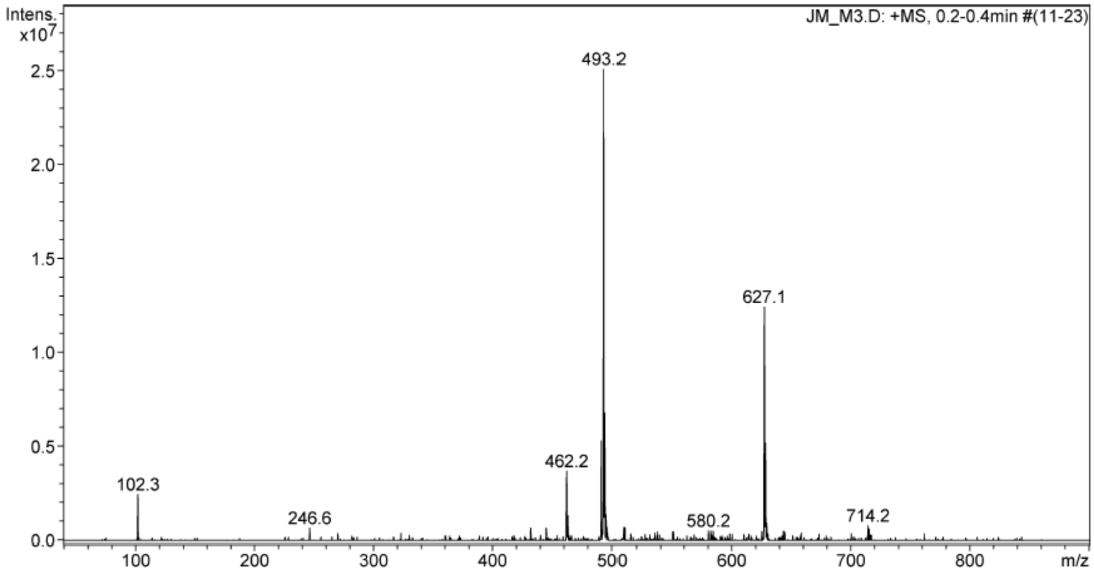

Figure S30. ESI-MS of **3ac**

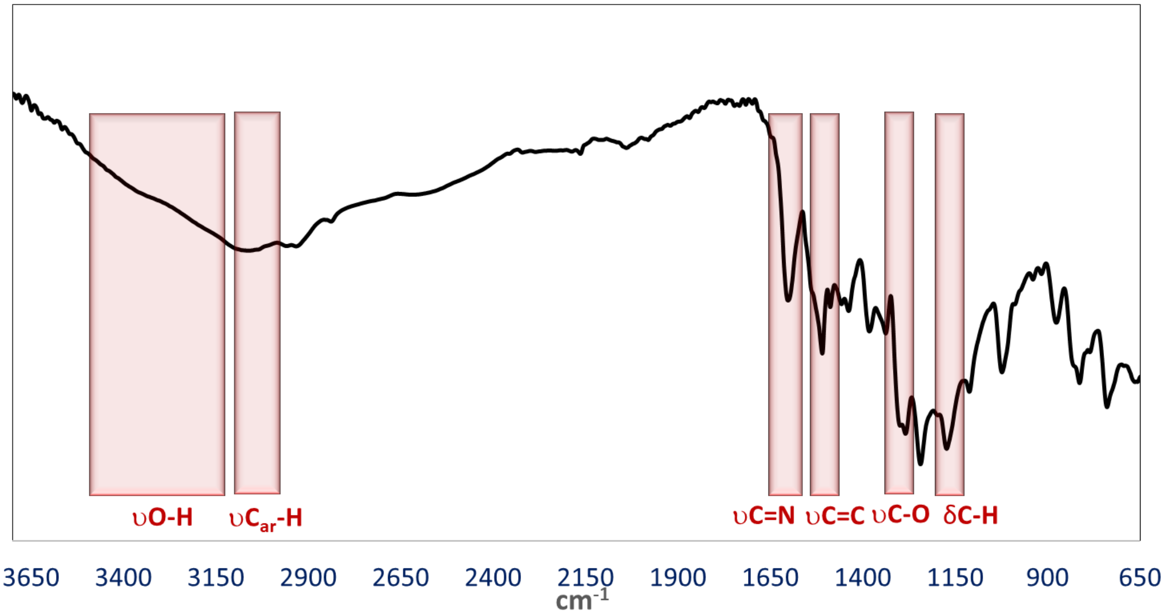

Figure S31. IR of **3ac**

2.5 Product 3ba

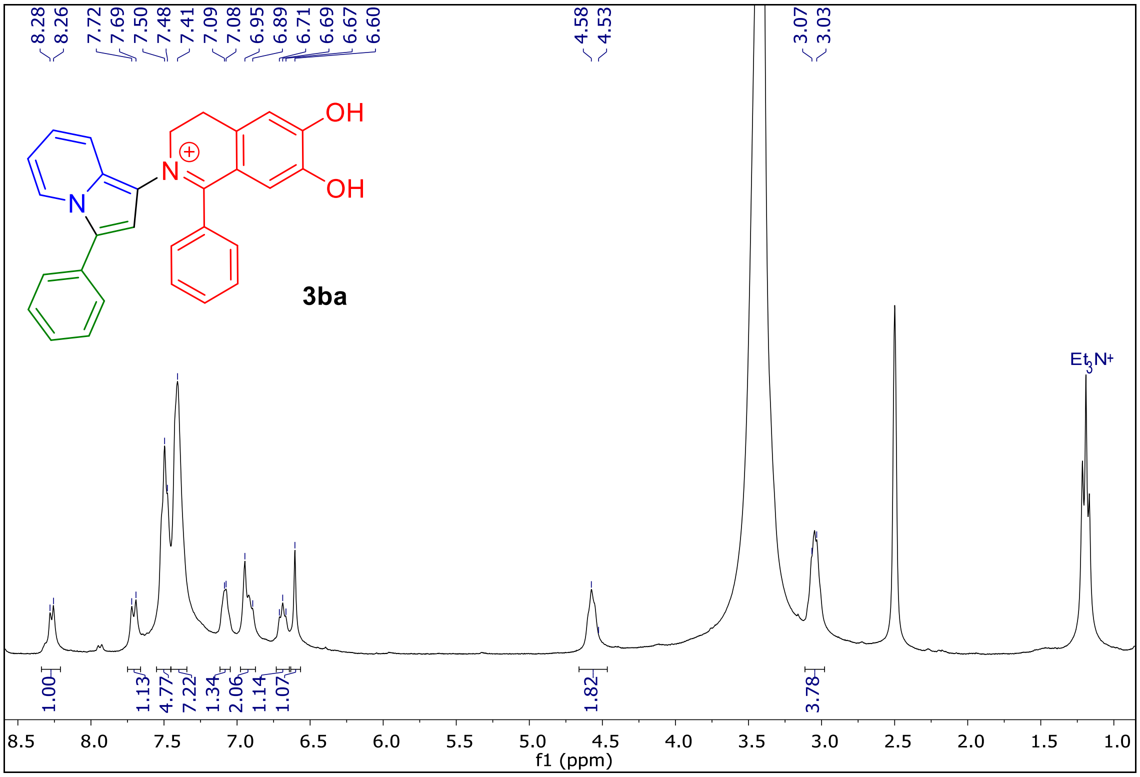

Figure S32. ^{1}H NMR of **3ba** in DMSO-d_6

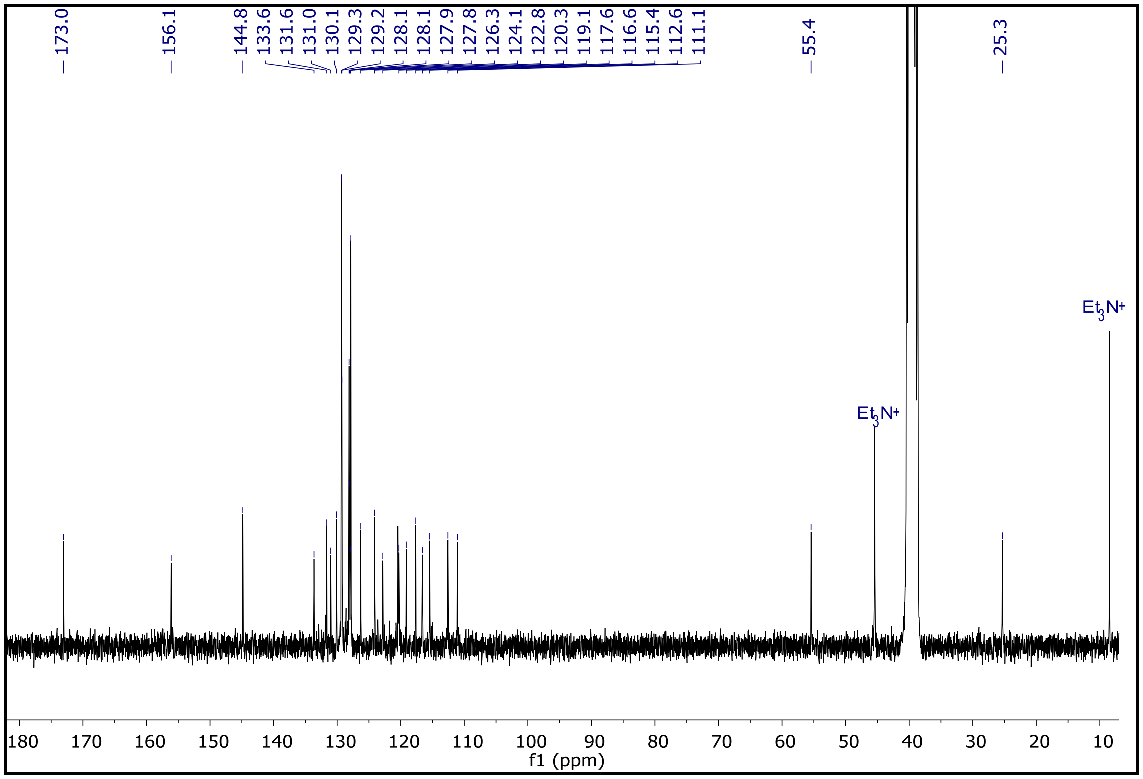

Figure S33. ^{13}C NMR of **3ba** in DMSO-d_6

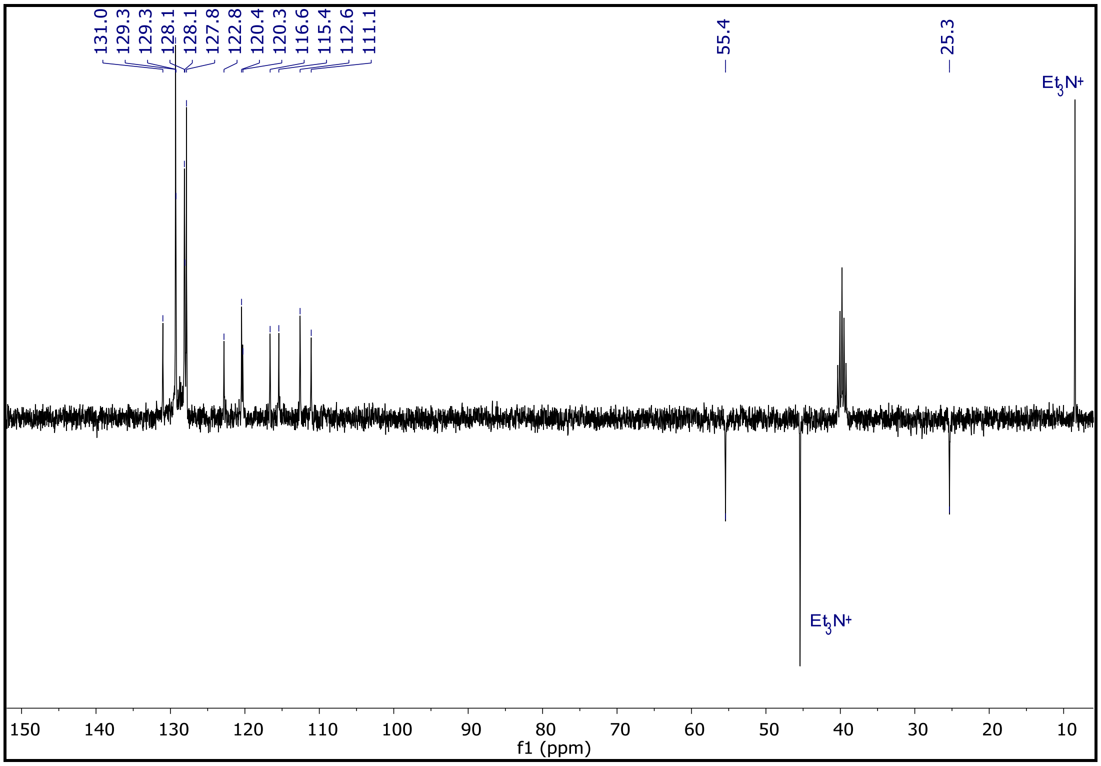

Figure S34. DEPT of **3ba** in DMSO-d$_6$

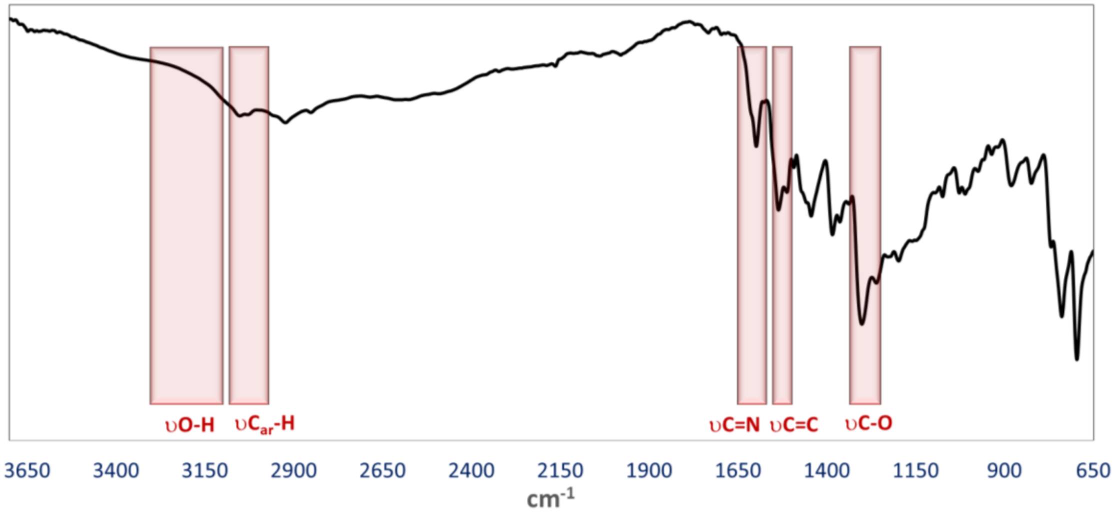

Figure S35. IR of **3ba**

3 XPS analysis

X-ray photoelectron spectroscopy was applied to characterize the sample surface state and composition. **Figure S36** shows the XP survey spectra for the C support, fresh CuNPs/C and post-reacted CuNPs/C samples.

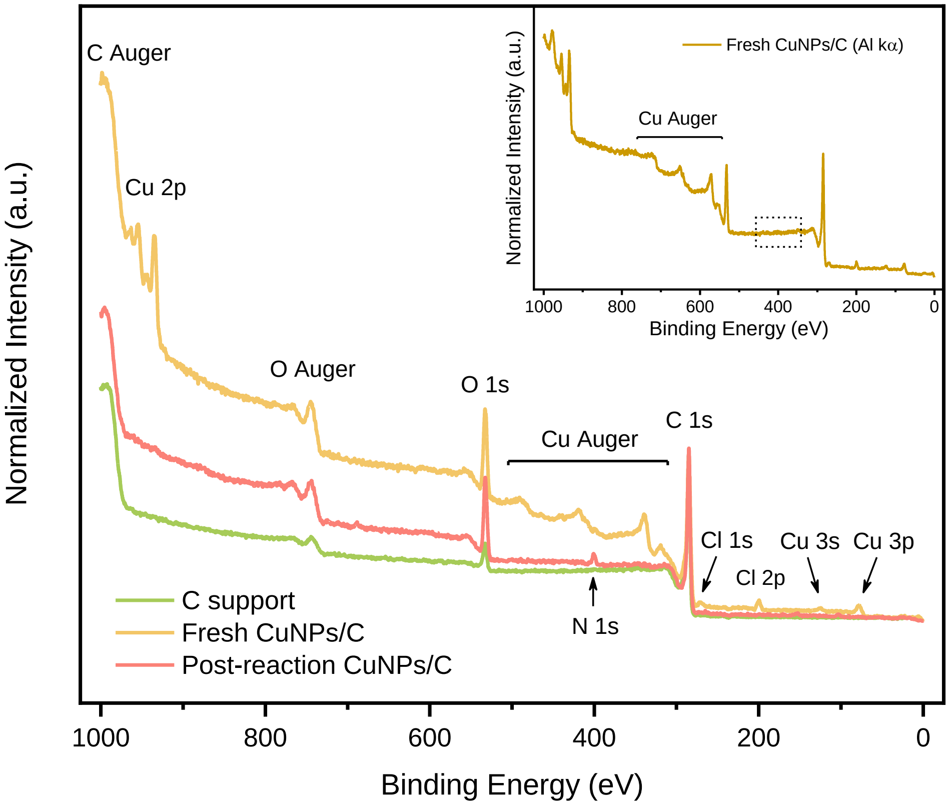

Figure S36 XP survey spectra normalized at the C 1s peak maximum intensity for the three analyzed samples, as indicated. The different labels point out the most relevant spectra features associated whit the elements present at the surface of the samples. The inset shows the survey spectrum taken for the fresh CuNPs/C sample whit the Al Kα anode radiation to avoid the overlap of the Cu LMM Auger structure whit the N 1s region (dash box).

Since C is the dominating element, the spectra were normalized by the maximum intensity of the corresponding C 1s peak for better comparison. As expected, signals from C, O, and Cu were detected by XPS. For the fresh nanocatalyst, a signal from Cl was also found probably be associated with the metallic precursor's residues. A noticeable decrease in the Cu signal could be observed for the post-reaction studied sample accompanied by the appearance of N. Because at this region also appears the wide structure coming from the Cu Auger LMM transitions, the fresh nanocatalyst was also analyzed with the Al Ka radiation (see inset at **Figure S36**). This could confirm that N appears as part of the reaction product and is not present in the C support or fresh CuNPs/C sample.
To get more insight into the nature and evolution of the surface species, XP resolution spectra deconvolutions were conducted for C support, fresh CuNPs/C, and post-rection CuNPs/C samples. **Figure S37** shows the C 1s and O 1s regions (left and right panels respectively). The C1s spectra were deconvoluted into five synthetic components. The main peak at 284.8 eV is assigned to sp2-hybridized graphite-like carbon (C-C) and the peaks centered at 286.0, 287.6, and 289.4 eV were attributed to

surface oxygen groups (designated as C-O, C=O and O-C=O, respectively), and the additional feature at about 292 eV is a satellite of sp2 graphite-like carbon in good agreement with the literature (Nagaiah et al., 2012; Sánchez et al., 2012; Khabashesku et al., 2000; Schlögl, 2008).

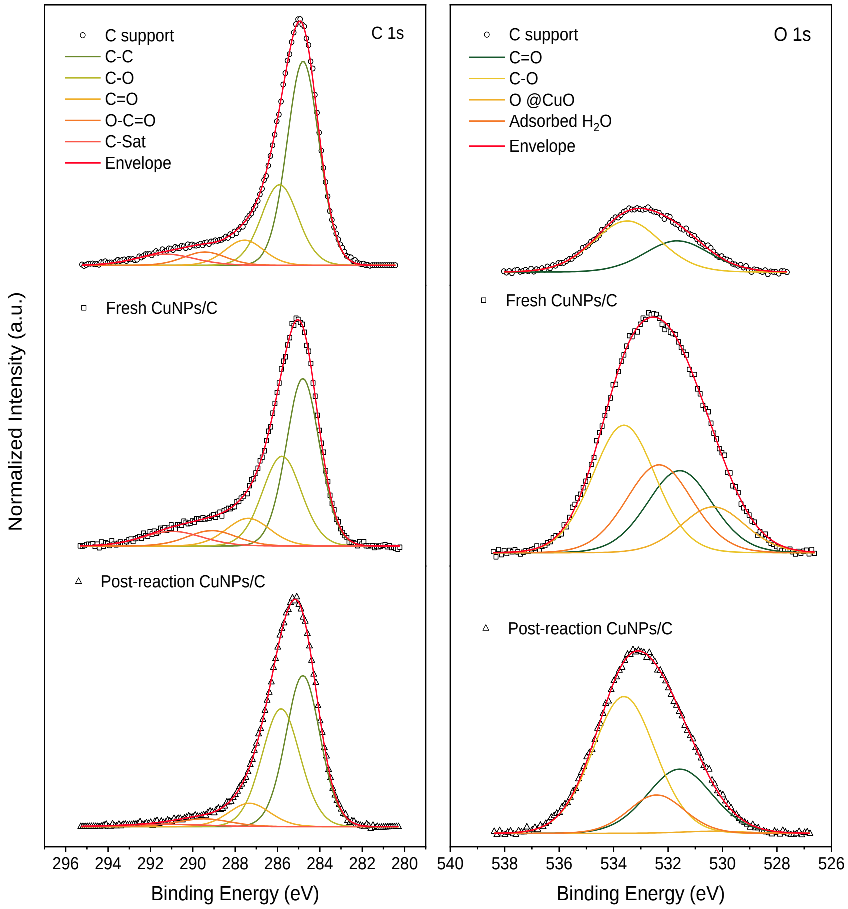

Figure S37. XP resolution spectra normalized to the C 1s total area for C 1s and O 1s region, respectively.

The O 1s region for the C support could be deconvoluted into two contributions at 531.6 and 533.6 eV indicating the presence of oxygen-containing functional groups with doubly and singly-bound oxygen (Schlögl, 2008). Besides these, the fresh and post-reaction nanocatalyst exhibit two additional peaks one at 530.3 eV, attributed to metal oxide, and the other at 532.3 eV associated with water or OH groups adsorbed on C during the CuNPs impregnation prosses.

Figure S38 (right panel) shows the Cu 2p XP resolution spectra. The Cu 2p spin-orbit coupled structure (Cu $2p_{3/2}$ and Cu $2p_{1/2}$) for the fresh nanocatalyst was well resolved by using three doublets at Cu $2p_{3/2}$ centered at 934.6 eV for CuO, 936.8 eV for $CuCl_2$ and 932.7 eV for Cu^0. Additional contributions were used to fit the shake-up satellite structure characteristics of the Cu^{2+} species (Biesinger, 2017). Considering the binding energies found for oxygen spices (**Figure S37**, right panel) and the characteristic shapes of the shake-up structure we discard the formation of Cu_2O and $Cu(OH)_2$.

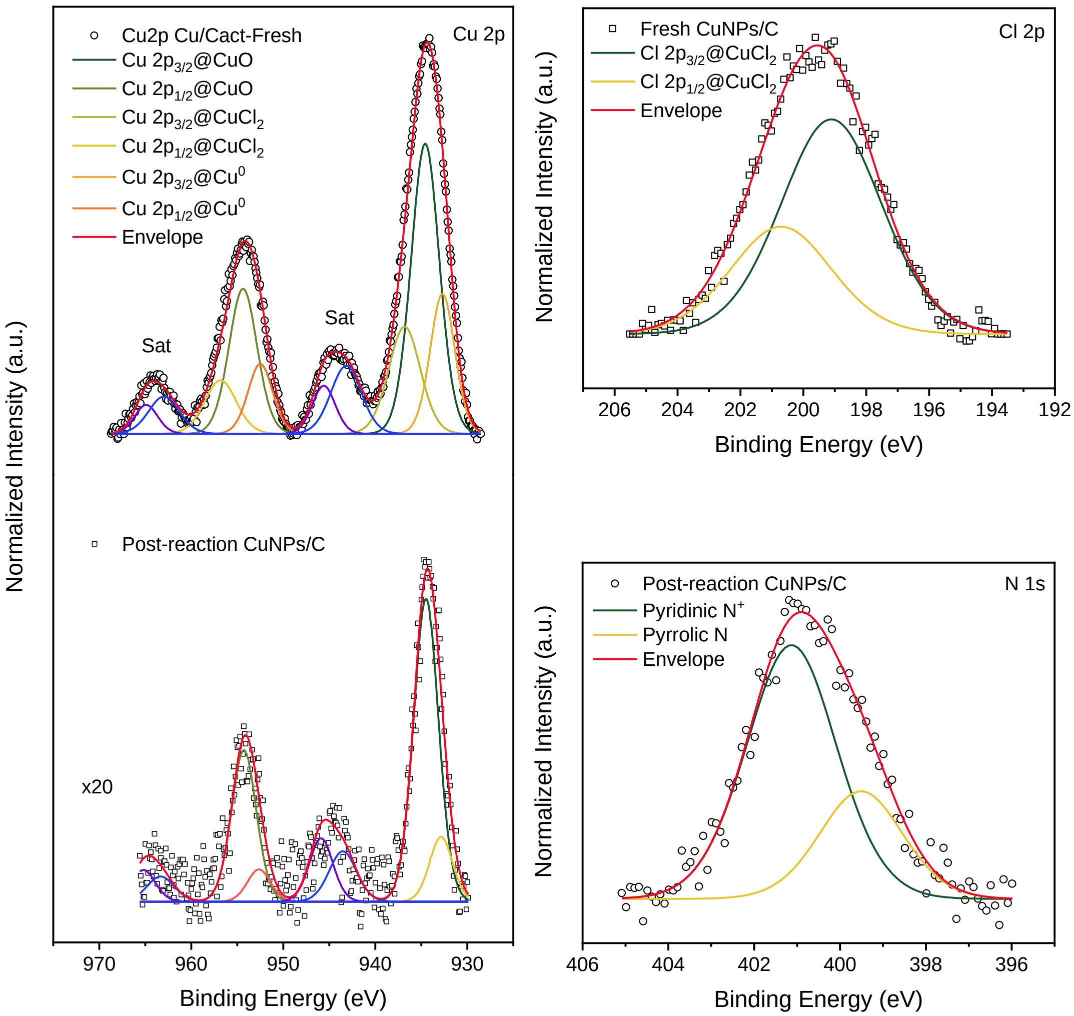

Figure S38. XP resolution spectra normalized to the C 1s total area for Cu 2p, Cl 2p, and N 1s region, respectively.

As already mentioned, the presence of $CuCl_2$ is justified by the Anhydrous copper (II) chloride used as a metal precursor and also was corroborated by XPS with a binding energy of 199.1 eV for the Cl $2p_{3/2}$, in good agreement whit the ones reported elsewhere for this compound (Biesinger, 2017).

Notwithstanding the fact of the remarkable decrease of the Cu 2p signal observed in the survey analysis (**Figure S36**), a resolution scan for the post-reaction sample could be obtained. As observed in Figure S37 (left panel), and despite the poor signal-to-noise relation, the spectrum could be deconvoluted into two contributions corresponding to CuO and Cu^0. The decrease in the Cu signal could be associated with the formation of capping substances coming from the reaction products as evidenced by the presence of N species (**Figure S38**, right bottom panel). The N 1s region was fitted using two

contributions at 401.1 eV and 399.5 eV as N-oxide pyridinic and pyrrolic nitrogen species, respectively (Nagaiah et al., 2012).

Table S1 summarised the surface atomic concentration derived from the XPS analysis.

Tabla S3.I: Surface atomic concentration obtained from deconvoluted XP spectra

	Surface Atomic Concentration (%)												
	C 1s				O 1s			N 1s		Cu 2p			Cl 2p
Sample	C-C	C-O	C=O	O-C=O	C=O	C-O	CuO	Pyrrolic N	Pyridinic N^+	CuO	$CuCl_2$	Cu0	$CuCl_2$
C support	56	25	8	5	5	4		-	-	-	-	-	-
Fresh CuNPs/C	49	19	7	4	4	6	2	-	-	2	1	1	2
Post-reaction CuNPs/C	37	32	7	3	4	8	0.1	1	2	0.1	-	0.03	-

4 UV-Vis spectra and fluorescence

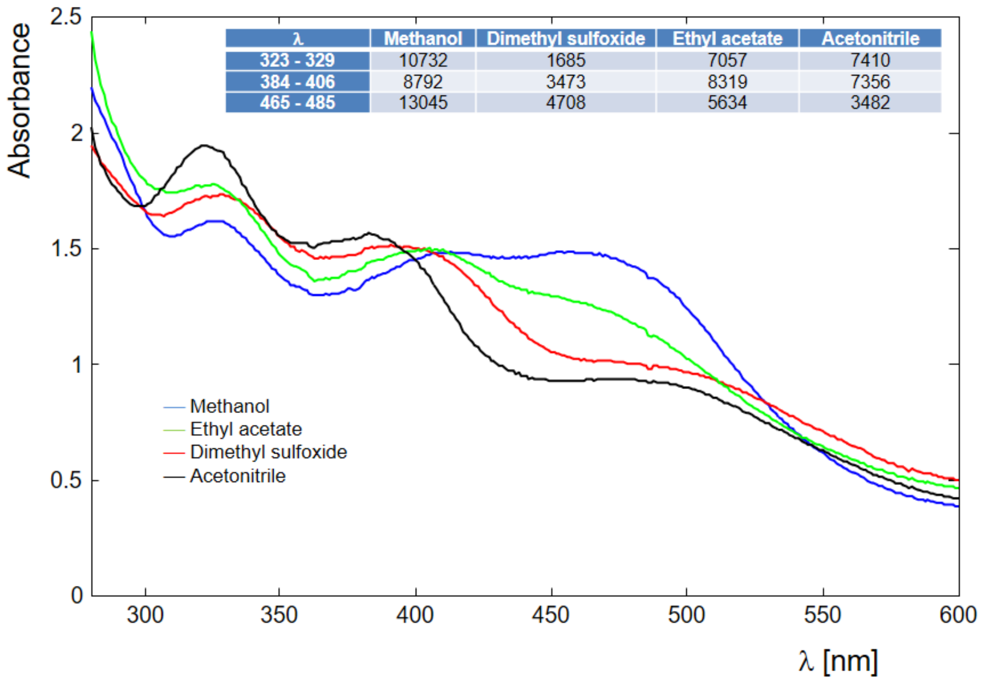

Figure S39. UV-Vis spectra of 3aa in different organic solvents.
The inserted table shows the absorption coefficients of 3aa in different solvents

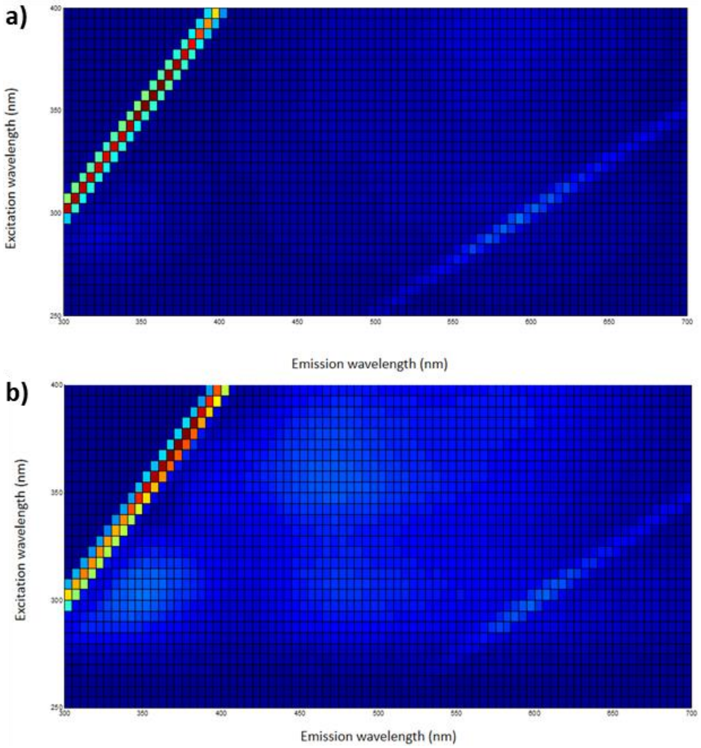

Figure S40. Excitation-Emission fluorescence of **3aa** in a) ACN and b) DMSO

•

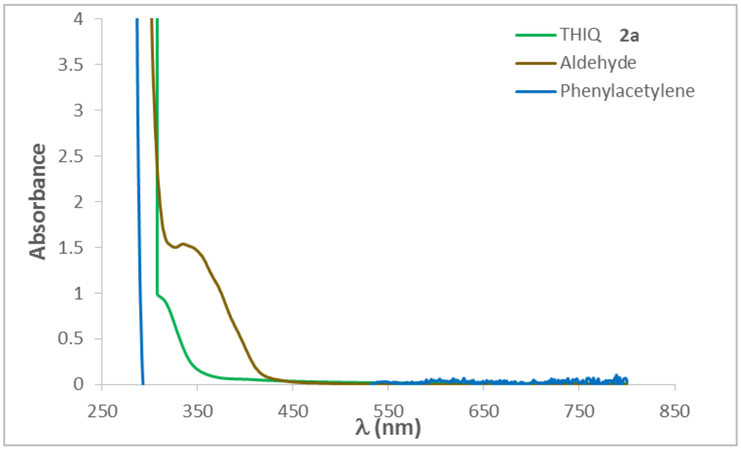

•

Figure S41. UV-Vis spectra of starting materials from product **3aa** in MeOH.

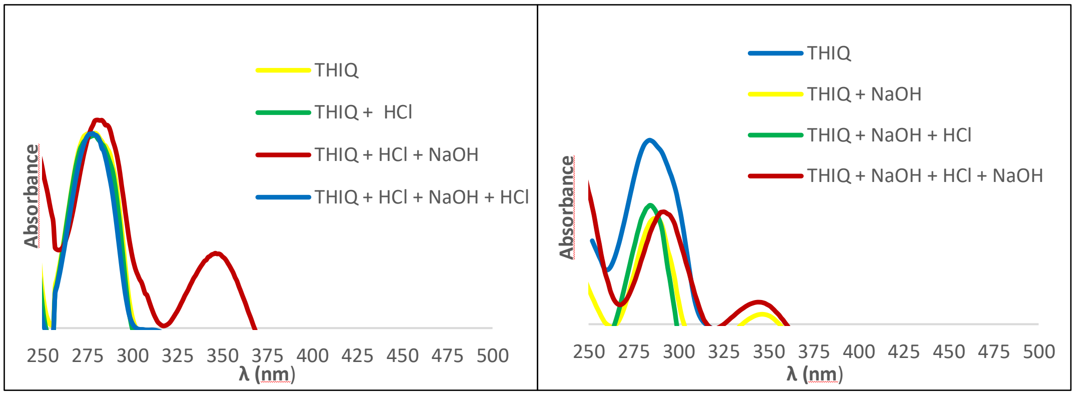

Figure S42. UV-Vis spectra for THIQ **2a** in HCl 0.1 M and NaOH 0.1 M solutions.

5 Comparison of ^{13}C-NMR of 3aa and 3ba

The most acidic OH group of **3ba** and **3aa** structures are probably those located at C6 and C6/C4' respectively, because of the resulting phenoxide ion after deprotonation, would be more stabilized in these cases (**Figure S43a**). Furthermore, ^{13}C RMN spectrum of **3ba** in DMSO-d$_6$/HCl shows only two C-OH (C6 and C7) at 156 ppm and 145 ppm. These two C-OH (C6 and C7) are found in the ^{13}C RMN spectrum of **3aa** practically in the same positions (**Figure S43b**).
Finally, to complete the proposed assignment of the C6 and C7 in **3aa**, we observe that when going from a DMSO-d$_6$/HCl medium to a DMSO-d6 medium, the C-OH signal that changes the most is the one located at 156 ppm, so it could correspond to the C6 because it is the one that undergoes the first deprotonation.

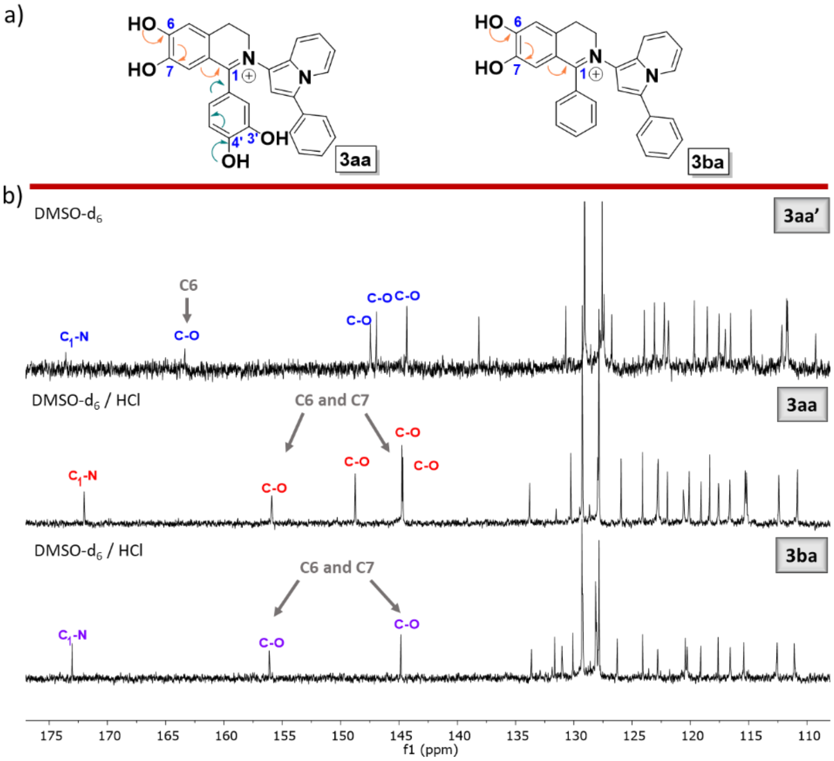

Figure S43. a) Structures of **3aa** and **3ba** products. b) ^{13}C-NMR spectra of **3aa, 3aa'** and **3ba** in different media. The proposed C6 assignment is also shown.

6 ^{13}C-NMR of 3 in different media

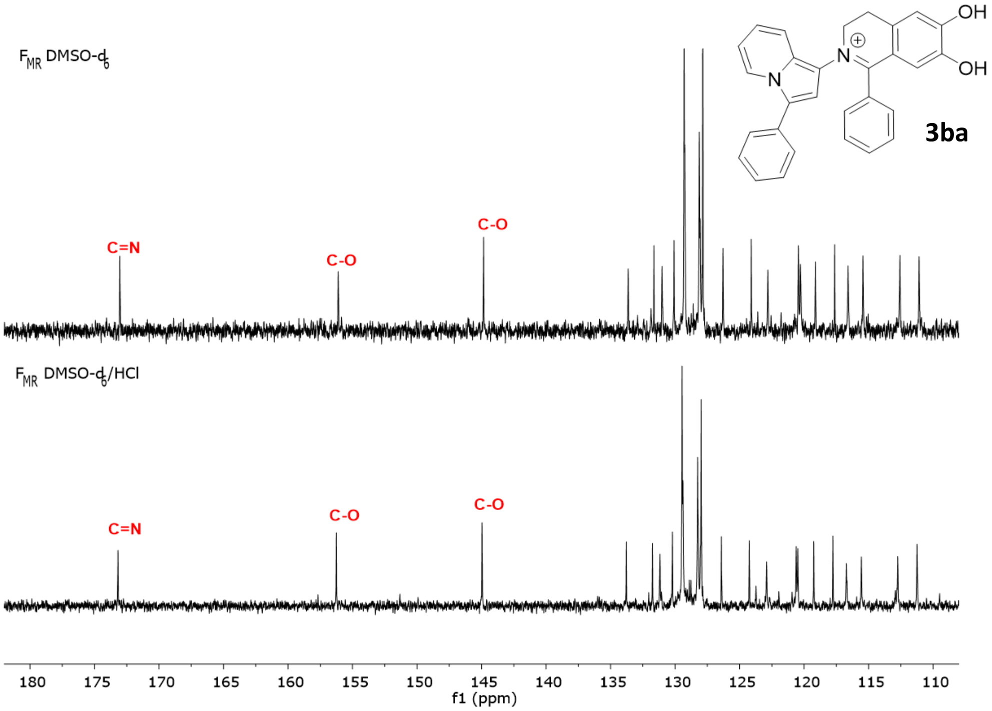

Figure S44. ^{13}C NMR spectra of **3ba** F_{MR} DMSO-d_6 and DMSO-d_6/HCl

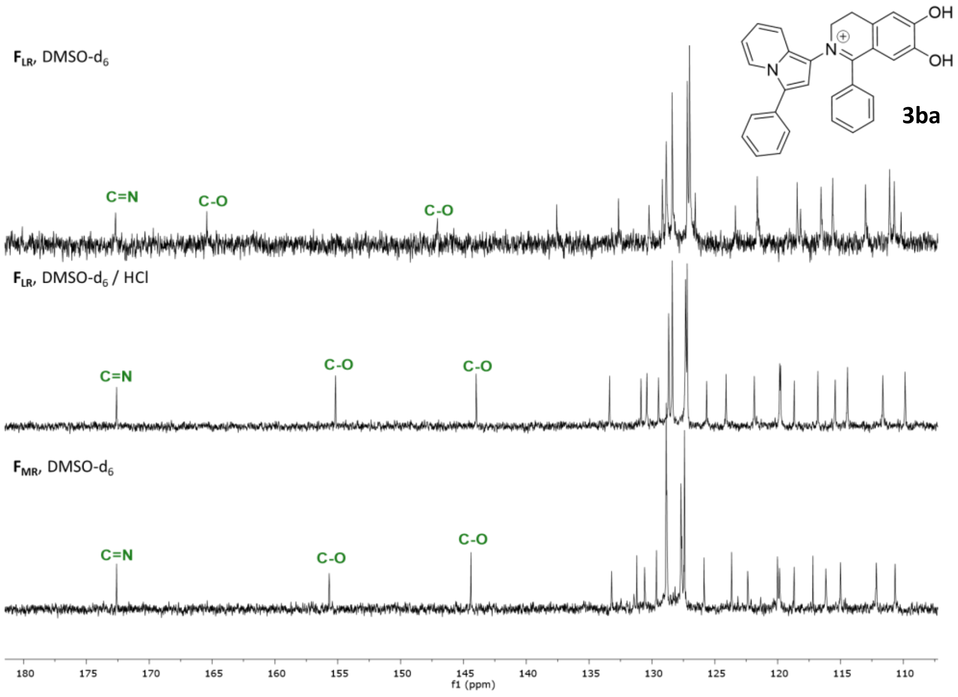

Figure S45. ^{13}C NMR spectra of **3ba** F_{LR} DMSO-d_6, F_{LR} DMSO-d_6/HCl and F_{MR} DMSO-d_6

7 References

Biesinger, M. C. (2017). Advanced analysis of copper X-ray photoelectron spectra. *Surf. Interface Anal.* 49(13), 1325-1334.

Khabashesku, V. N., Zimmerman, J. L., Margrave, J. L. (2000). Powder Synthesis and Characterization of Amorphous Carbon Nitride. *Chem. Mater.* 12, 3264-3270.

Nagaiah, T. C., Bordoloi, A., Sánchez, M. D., Muhler, M., Schuhmann, W. (2012). Mesoporous Nitrogen-Rich Carbon Materials as Catalysts for the Oxygen Reduction Reaction in Alkaline Solution. *ChemSusChem*, 5(4), 637-641.

Sánchez, M. D., Chen, P., Reinecke, T., Muhler, M., Xia, W. (2012). The Role of Oxygen- and Nitrogen-containing Surface Groups on the Sintering of Iron Nanoparticles on Carbon Nanotubes in Different Atmospheres. *ChemCatChem.* 4(12), 1997-2004.

Schlögl, R. (2008). "Carbons" in Handbook of heterogeneous catalysis, eds. G. Ertl, H. Knözinger, F. Schüth, and J. Weitkamp (Wiley VCH Verlag, Weinheim), 357-427.
